# Sulforaphane elicts dual therapeutic effects on Renal Inflammatory Injury and crystal deposition in Calcium Oxalate Nephrocalcinosis

**DOI:** 10.7150/thno.44054

**Published:** 2020-06-05

**Authors:** Haoran Liu, Xiaoqi Yang, Kun Tang, Tao Ye, Chen Duan, Peng Lv, Libin Yan, Xiaoliang Wu, Zhiqiang Chen, Jianhe Liu, Yaoliang Deng, Guohua Zeng, Jinchun Xing, Zhangqun Ye, Hua Xu

**Affiliations:** 1 Department of Urology, Tongji Hospital, Tongji Medical College, Huazhong University of Science and Technology, Wuhan, China.; 2 Department of Urology, The Second Affiliated Hospital of Kunming Medical University, Kunming, China.; 3 Department of Urology, The First Affiliated Hospital of Guangxi Medical University, Nanning, China.; 4 Department of Urology, The First Affiliated Hospital of Guangzhou Medical University, Guangzhou, China.; 5 Department of Urology, The First Affiliated Hospital of Xiamen University, Xiamen, China.

**Keywords:** Sulforaphane, Nrf2, TLR4, IRF1, Macrophage

## Abstract

Intrarenal calcium oxalate (CaOx) crystals induce renal tubular epithelial cells (TECs) injury and inflammation, which involve Toll-like receptor 4 (TLR4)/interferon regulatory factor 1 (IRF1) signaling. Additionally, infiltrating macrophages (Mϕs) might influence intrarenal CaOx crystals and CaOx-induced renal injury. Although the roles of nuclear factor erythroid 2-related factor 2 (Nrf2) in regulating inflammation and macrophage polarization are well characterized, its potential mechanisms in regulating CaOx nephrocalcinosis remain undefined.

**Methods:** We used a Gene Expression Omnibus dataset to analyze gene-expression profiles. Luciferase reporter, western blot, quantitative polymerase chain reaction, immunofluorescence staining, fluorescence in situ hybridization, positron emission tomography computed tomography imaging, flow cytometry, and chromatin immunoprecipitation assays were employed to study the mechanism of miR-93-TLR4/IRF1 regulation by Nrf2. Anti-inflammatory activity and regulation of macrophage polarization by Nrf2 were investigated *in vitro* and *in vivo*.

**Results:** We found that stone-mediated kidney inflammation significantly affected stone growth, and that sulforaphane attenuated CaOx nephrocalcinosis-induced kidney injury and renal CaOx crystals deposition. Additionally, Nrf2 levels significantly increased and negatively correlated with TLR4 and IRF1 levels in a mouse model of CaOx nephrocalcinosis following sulforaphane treatment. Moreover, Nrf2 suppressed TLR4 and IRF1 levels and decreased M1-macrophage polarization which induced by supernatants from COM-stimulated TECs *in vitro*. In terms of mechanism, transcription factor analyses, microRNA microarray, and chromatin immunoprecipitation assays showed that Nrf2 exhibited positive transcriptional activation of miR-93-5p. In addition, Luciferase reporter, qRT-PCR, and western blot validated that miR-93-5p targets TLR4 and IRF1 mRNA. Furthermore, suppressed miR-93-5p expression partially reversed Nrf2-dependent TLR4/IRF1 downregulation.

**Conclusions:** The results suggested that sulforaphane might promote M2Mϕ polarization and inhibit CaOx nephrocalcinosis-induced inflammatory injury to renal tubular epithelial cells via the Nrf2-miR-93-TLR4/IRF1 pathway *in vitro* and *in vivo.*

## Introduction

Kidney stone disease affects approximately 9% of adults worldwide during their lifetime, with this number continuing to increase 1, 2. Calcium oxalate (CaOx) stones account for >80% of all types of kidney stones and can cause nephrocalcinosis 3. Different from symptomatic urolithiasis, CaOx nephrocalcinosis is commonly asymptomatic but can cause serious intrarenal inflammation and kidney tubular cell necroptosis, thereby increasing CaOx crystal adhesion 4.

Calcium oxalate stones can be originated from supersaturation of calcium and oxalate ions, leading to crystallization inside renal tubular 5, 6. Then the deposition of “stone nidus” on tubular cell membranes is promoted by the absence of crystallization inhibitors and the presence of proteins that facilitate crystal adhesion 7-10. Crystals are known to be cytotoxic and injurious when in direct contact with the tubular epithelium 11. Internalized crystals have been demonstrated to induce tubular cell necrosis by altering mitochondrial function, leading to increased reactive oxygen species production, lysosomal destabilization and protease leakage, and activation of the NLRP3 inflammasome 12-15. Necrotic cells release numerous damage-associated molecular patterns (DAMPs) such as ATP, histones or HMGB1 and alarmins. These particles activate Toll-like receptors and trigger the formation of inflammasomes in dendritic cells and pro-inflammatory macrophages, which amplify renal inflammation and contribute to crystal-induced AKI 5, 7, 16, 17.

Macrophages (Mϕs) are crucial innate immune cells that can be induced into functionally distinct phenotypes under various stimuli 18. M1Mϕ exerts proinflammatory effects and facilitates the progression of chronic kidney disease, whereas M2Mϕ exerts anti-inflammatory and tissue-healing effects that limit nephropathy 19-21. Data from several studies suggest that M1Mϕ might contribute to crystal deposition by promoting inflammation-related oxidative stress, but that M2Mϕ eliminates crystal deposition by phagocytosis 22, 23.

Sulforaphane (SFN) is isolated from cruciferous vegetables and a pharmacological activator of nuclear factor erythroid 2-related factor 2 (Nrf2), which promotes anti-inflammatory responses 24, 25. Nrf2 is a key factor involved in physiological and pathological anti-inflammatory and pro-survival processes 26. A recent study indicated that Nrf2 might regulate M1Mϕ/M2Mϕ polarization in the liver by affecting peroxisome proliferator-activated receptor-γ expression 27. Another report showed that lactate derived from cancer cells drives M2Mϕ polarization by activating Nrf2 28. However, the roles of Nrf2 signaling in regulating the phenotypes of infiltrating Mϕ to influence intrarenal crystals elimination remain largely unknown.

Toll-like receptor 4 (TLR4) is a critical pattern-recognition receptor in the host-defense system and a key regulator of Mϕ polarization to M1 29. After recognizing a pathogen and recruiting the adaptor protein MyD88, TLR4 triggers nuclear factor kappaB (NF-κB) transcription, which activates several signaling pathways that regulate Mϕ functions, including phagocytosis, and increases levels of proinflammatory cytokines [e.g., inducible nitric oxide synthase (iNOS), interleukin (IL)-1β, cyclooxygenase-2, IL-6, and tumor necrosis factor (TNF)-α] directly involved in kidney injury 30, 31. Interferon regulatory factor 1 (IRF1) interacts with signal transducer and activator of transcription 1, a transcription factor critical to M1Mϕ polarization, and promotes proinflammatory cytokine production 32, 33. Our previous data demonstrated that IRF1 participates in renal crystal formation and inflammatory injury by regulating the Mϕ-specific immune responses 34.

In the present study, we investigated the effects of SFN on CaOx-induced nephrocalcinosis 35. Our findings highlighted the underlying novel mechanisms, whereby the Nrf2-miR-93-TLR4/IRF1 axis suppresses CaOx nephrocalcinosis-induced renal injury and promotes crystal elimination by regulating Mϕ polarization. The results suggest that SFN can potentially serve as a preventative and therapeutic drug for CaOx-induced nephrocalcinosis.

## Materials and methods

### Animal procedures

C57BL/6J mice (6-8-weeks old) were provided by the Experimental Animal Research Center of Hubei (SCXK: 2017-0106; Wuhan, China) and raised in the Animal Facilities of Tongji Hospital under pathogen-free conditions. All animals were raised according to the NIH Guide for the Care and Use of Laboratory Animals. To establish the murine model of CaOx nephrocalcinosis, each mouse received intraperitoneal injection with vehicle (saline) or glyoxylate(Gly; 75 mg/kg/d; 200 μL) on days 4 through 10 35. Mice in the intervention groups received intraperitoneal injection of SFN (MedChemExpress, Monmouth Junction, NJ, USA) at increasing concentrations (10, 25, and 50 mg/kg/d; 200 μL) on days 1 through 10. A long-acting miR-93 antagonist (antagomir-93) was synthesized by RiboBiotech (Guangzhou, China). Mice received AntagomiR-93 (20 mg/kg; 200 µL) through the tail vein on days 1, 4, and 7. Ten days later, all animals were sacrificed, and renal samples were collected and fixed in 4% paraformaldehyde. This study was approved by the Ethics Committee of Tongji Hospital, Huazhong University of Science and Technology.

### Cell culture and co-culture

The tibias and femurs of C57BL/6 mice were excised and washed with ice-cold sterile phosphate-buffered saline (PBS) to obtain bone marrow-derived macrophages (BMDMs). BMDMs were maintained in RPMI 1640 (Sigma-Aldrich, St. Louis, MO, USA) supplemented with 10% fetal bovine serum (Sigma-Aldrich) and 50 ng/mL macrophage colony-stimulating factor (BD Biosciences, Franklin Lakes, NJ, USA) at 37°C and 5% CO_2_ for 1 week. On day 5, non-adherent cells were discarded, leaving adherent cells for subsequent experiments.

Renal tubular epithelial cells (TECs) were isolated from C57BL/6 mouse renal tissues. Briefly, the renal cortex was fragmented, and the TECs were dissociated from the renal cortex by digestion with 1 mg/mL type I collagenase for 30 min at 37°C, followed by isolation using gradient-density centrifugation in Percoll. TECs purity was measured by immunostaining for cytokeratin-18 and staining with Hoechst dye.

To better study functional changes of macrophage in response to soluble mediators released from calcium oxalate monohydrate (COM) stimulated TECs; we build up BMDMs-COM-stimulated TECs co-culture system. BMDMs were seeded in the lower chamber of Transwell plates with a 0.4-mm pore size (Corning, Inc., Corning, NY, USA), and TECs were seeded in the upper chamber treated with 100 μg/mL COM and incubated for 24 h 36.

### Chemicals and materials

Four synthetic microRNA (miRNA) oligonucleotides (i.e., an miR-93-5p mimics, miR-93-5p inhibitor, negative-control mimics, and negative-control inhibitor) were obtained from RiboBiotech. A negative-control small-interfering RNA (siRNA) and a Nrf2 siRNA [siNrf2; 5′-UUGGGAUUCACGCAUAGGAGCACUG-3′ (sense)] were purchased from RiboBiotech. The miRNA oligonucleotides and siNrf2 were transfected with riboFECT CP according to manufacturer instructions (RiboBiotech).

### Detection of kidney CaOx crystals

Kidney sections were stained with hematoxylin-eosin (HE) according to standard procedures and visualized using a polarized light optical microscope (Zeiss, Oberkochen, Germany). Additionally, kidney sections were stained using the Pizzolato method to determine crystal deposition 37. Kidney crystals were quantified using ImageJ software (National Institutes of Health, Bethesda, MD, USA).

### Tubular injury assessment

Slices of renal tissues were dyed with periodic acid-Schiff (PAS) to evaluate tubular injury, including tubular dilation, tubular atrophy, tubular cast formation, sloughing of tubular epithelial cells, or thickening of the tubular basement membrane 38. Ten non-overlapping microscopic fields (200×) were randomly selected, and the percentage of cells with tubular injury among total TECs was calculated (scoring: none, 0; <25%, 1; 25-50%, 2; 51-75%, 3; and >75%, 4) 38. The average score was calculated to represent the average injuried level of the 10 fields examined. Terminal deoxynucleotidyl transferase dUTP nick-end labeling (TUNEL) staining was performed to assess renal cell death using an in situ cell detection kit (Roche, Rotkreuz, Switzerland) 39. Positive cells in TUNEL assays were counted in 10 randomly chosen magnification fields (200×) for each slice.

### Immunohistochemistry (IHC)

Renal samples were fixed with formalin and embedded in paraffin for routine sectioning, followed by HE staining. For IHC, slices were incubated with anti-Nrf2 (1:400; Affinity Biologicals, Cincinnati, OH, USA), anti-TLR4 (1:1800; Servicebio, Wuhan, China), and anti-IRF1 (1:1000; Absin, Shanghai, China) overnight at 4°C, followed by evaluation using an Envision HRP Polymer system (Boster, Wuhan, China) and a Leica SCN400 scanner (Leica Biosystems, Wetzlar, Germany). Relative expression levels were analyzed using ImageJ software (National Institutes of Health).

### Measurement of blood urea nitrogen (BUN) and serum creatinine

Blood samples were taken from mice on days 3, 4, and 10. Serum levels of BUN and creatinine were determined using commercial kits (Stanbio Laboratory, Boerne, TX, USA).

### Fluorescence in situ hybridization (FISH)

A FAM-labeled mmu-miR-93-5p nucleic acid probe and a FISH kit were obtained from RiboBiotech, with experiments conducted according to manufacturer instructions. All images were acquired using a fluorescence microscope (Olympus, Tokyo, Japan) equipped with a digital camera.

### Positron emission tomography-computed tomography (PET-CT) imaging

^18^F-FDG is an analog of deoxyglucose, which tends to accumulate in tissues that consume high levels of glucose. Activated inflammatory cells with high glucose consumption also show high ^18^F-FDG uptake 40. Recent studies assessed renal inflammation and acute renal injury by ^18^F-FDG PET-CT 41-43. In the present study, 150 subjects diagnosed with lung cancer and who had undergone the same radiotherapy treatment were retrospectively studied in Tongji Hospital from January 2016 to March 2017. This study was approved by the Ethics Committee of Tongji Hospital, Huazhong University of Science and Technology. Ten patients were found to have renal stones according to an initial PET-CT scan. After 6 months, PET-CT was used to explore the intensity of ^18^F-FDG uptake around the renal parenchyma. Standardized uptake-value (SUV) measurements were acquired for the region of interest (ROI). To further eliminate the residual radionuclide signals in urine, the GE Segment threshold model was used to remove renal pelvis. All PET-CT images were evaluated by three certified radiologists in consensus.

For animal experiments, each animal was injected with 200 ± 10 μCi ^18^F-FDG via the caudal vein. After 1 h, mice were anesthetized with 2% isoflurane for PET scanning, which was performed using a static scanning pattern (10 min) with the Trans-PET BioCaliburn 700 System (Raycan Technology Co., Ltd., Suzhou, China). Images were reconstructed using the three-dimensional ordered subset expectation-maximization method (voxel size: 0.5 × 0.5 × 0.5 mm^3^). Volume-of-interest analysis was performed using AMIDE software (Free Software Foundation, Inc., Boston, MA, USA). Mean SUV was calculated as: Mean SUV = mean pixel value of the decay corrected ROI (μCi/kg) / (injected dose [μCi] / weight [kg]).

### Enzyme-linked immunosorbent assay (ELISA)

Cytokine levels in the supernatants of co-cultured cells were determined using ELISA kits for IL-1β (DY401; R&D Systems, Minneapolis, MN, USA), TNF-α (DY410; R&D Systems, Minneapolis, MN, USA) IL-6 (BMS603-2; Thermo Fisher Scientific, Waltham, MA, USA), and IL-10 (DY417; R&D Systems, Minneapolis, MN, USA). Serum cytokine levels were assessed using ELISA kits according to manufacturer instructions.

### Real-time quantitative polymerase chain reaction (qPCR)

Total RNA was extracted from BMDMs and reverse transcribed to acquire cDNA using TRIzol reagent (Invitrogen, Carlsbad, CA, USA) and the PrimeScript RT reagent kit (TaKaRa, Shiga, Japan), followed by qPCR amplification using SYBR Green master mix (Yeasen, Shanghai, China) according to a standard protocol. Relative gene expression was normalized against glyceraldehyde 3-phosphate dehydrogenase (GAPDH). Primer sequences are listed in Table S1. Mature miRNAs were quantitatively measured using an All-in-One miRNA qRT-PCR detection kit (GeneCopoeia, Rockville, MD, USA) and normalized against U6 RNA.

### Western blot

Total protein was extracted from BMDMs in radioimmunoprecipitation lysis buffer with a complete protease-inhibitor cocktail (Servicebio). Protein concentrations were detected using a BCA protein assay kit (Beyotime Biotechnology, Beijing, China). Protein extract was isolated by 10% sodium dodecyl sulfate polyacrylamide gel electrophoresis, transferred to polyvinylidene fluoride membranes, and incubated overnight at 4°C with primary antibodies against Nrf2 (AF7006; 72 kDa; 1:1000; Affinity Biologicals), TLR4 (GB11519; 95 kDa; 1:1000; Servicebio), IRF1 (abs118047; 37 kDa; 1:1000; Absin), iNOS (BA0362; 130 kDa; 1:200; Boster), arginase 1 (ARG-1; GB11285; 35-40 kDa; 1:5000; Servicebio), or GAPDH (T0004; 34 kDa; 1:5000; Affinity Biologicals). The membrane strips were then stained by incubating with an appropriate horseradish peroxidase-conjugated secondary antibody for 2 h at 25°C and visualized by enhanced chemiluminescence (Millipore, Billerica, MA, USA). ImageJ software (National Institutes of Health) was used to quantify the relative densities of proteins, which were normalized against GAPDH. All experiments were performed in triplicate.

### Immunofluorescence

BMDMs seeded on coverslips were fixed with 4% paraformaldehyde and permeabilized with 0.2% Triton X-100. Fixed cells were then blocked with goat serum and incubated with a primary antibody against ARG-1 (16001-1-AP; 1:200; Proteintech Group, Wuhan, China) or iNOS (610431; 1:200; BD Biosciences) at 4°C overnight, followed by staining with CY3- or Alexa Fluor-488-conjugated secondary antibodies (1:1000; Thermo Fisher Scientific). The slides then were stained with 4′,6-diamidino-2-phenylindole (DAPI) and mounted for analysis. All images were captured using a fluorescence microscope (Nikon, Tokyo, Japan) at the same settings.

Paraffin-embedded mouse kidney sections were deparaffinized for subsequent antigen retrieval. Kidney sections were then blocked with goat serum and incubated with a primary antibody against ARG-1 (sc-271430; 1:200; Santa Cruz Biotechnology, Dallas, TX, USA) or iNOS (BA0362; 1:200; Boster) at 4°C overnight, followed by staining with fluorescein isothiocyanate (FITC)- or CY3-conjugated secondary antibodies (1:1000; Thermo Fisher Scientific). Slides were stained with DAPI and mounted for analysis, and all images were captured using a fluorescence microscope (Nikon, Tokyo, Japan) at the same settings.

### Flow cytometry

To analyze M1Mϕ and M2Mϕ polarization, BMDMs were probed with antibodies against phycoerythrin (PE)-conjugated F4/80 (565410; BD Pharmingen, San Diego, CA, USA), FITC-conjugated CD11b (557396; BD Pharmingen), PE-Cy7-conjugated CD11c (558079; BD Pharmingen), and Alexa Fluor-647-conjugated CD206 (565250; BD Pharmingen) at room temperature. TECs were stained using the propidium iodide necrosis detection kit (eBioscience, San Diego, CA, USA). All stained cells were analyzed by flow cytometry (BD Biosciences) using FlowJo software (v.10.0; Tree Star, Ashland, OR, USA).

### COM crystals internalization by BMDMs

COM crystals were stained, as follows: 1 mg/mL COM crystal (C0350000; Sigma-Aldrich) (pH 7.4) was incubated for 3 h in the dark at 25°C with an Alexa Fluor-488-conjugated anti-rabbit IgG molecular probe (4412S; 1:400; Cell Signaling Technology, Danvers, MA, USA) at a concentration of 0.01 mg/mL. The labeled crystals were washed three times with PBS and collected by centrifugation at 1000g for 10 min. Firstly, treated BMDMs were co-culture with COM-stimulated TECs for 24 hours. Then, BMDMs were directly cultured with 100 µg/mL IgG Alexa Fluor-488-labeled COM crystals for 5 h. BMDMs were washed with PBS three times to eliminate unbound COM crystals 44, 45, and internalized crystal were observed under a fluorescence microscope (Nikon, Tokyo, Japan).

### Chromatin immunoprecipitation (ChIP) assay

To analyze direct activation of the miR-93-5p promoter by Nrf2, ChIP assays were performed using the EZ-Magna ChIP kit (Millipore). BMDMs were pretreated with Nrf2 overexpression plasmid for 48 h and fixed with 1% formaldehyde for 10 min at 37°C to cross-link DNA and proteins. Following termination of the cross-linking reaction with 0.125 M glycine, the BMDMs were washed twice with PBS by centrifugation at 800g and 4°C for 5 min. After cell lysis, the cross-linked chromatin was sheared into fragments (200-1000 bp) by sonication, and samples were immunoprecipitated with an anti-Nrf2 antibody (12721S; 1:200; Cell Signaling Technology). Normal mouse IgG antibodies were used as negative controls. DNA samples were purified using magnetic beads and then amplified by ChIP-qPCR using the following primers: F, 5′-TGTCTCGGTCTGACAGTG-3′; and R, 5′-GGAATGAAGTCAAGGATCTTTC-3′.

### Luciferase reporter assay

To construct luciferase reporter plasmids, the 3′ untranslated region (UTR) of possible target genes (IRF1 or TLR4) containing the putative binding site of miR-93-5p and the same region with a mutation in the miR-93-5p seed sequence were inserted into the psiCHECK2 vector. BMDMs were co-transfected with 50 nM miR-93-5p mimics or negative-control miRNA along with either the wild-type (WT) or mutant 3′ UTR reporter plasmid using Lipofectamine 3000 (Invitrogen). The Dual-Luciferase Assay System was used to assess luciferase activities at 48-h post-transfection. Relative expression levels were expressed in terms of firefly luciferase activity normalized against Renilla luciferase activity.

### Statistical analysis

GraphPad Prism software (v.5.0; GraphPad Software, La Jolla, CA, USA) was used for data analyses. Experimental data were presented as the mean ± standard deviation. Student's t test and one-way analysis of variance (ANOVA) were performed to compare differences between groups. Pearson's correlation test was applied to analyze correlations between genes. P < 0.05 was considered statistically significant.

## Results

### PET-CT showed higher ^18^F-FDG uptake and inflammatory status in rapidly progressive stone patients

**Figure 1:**
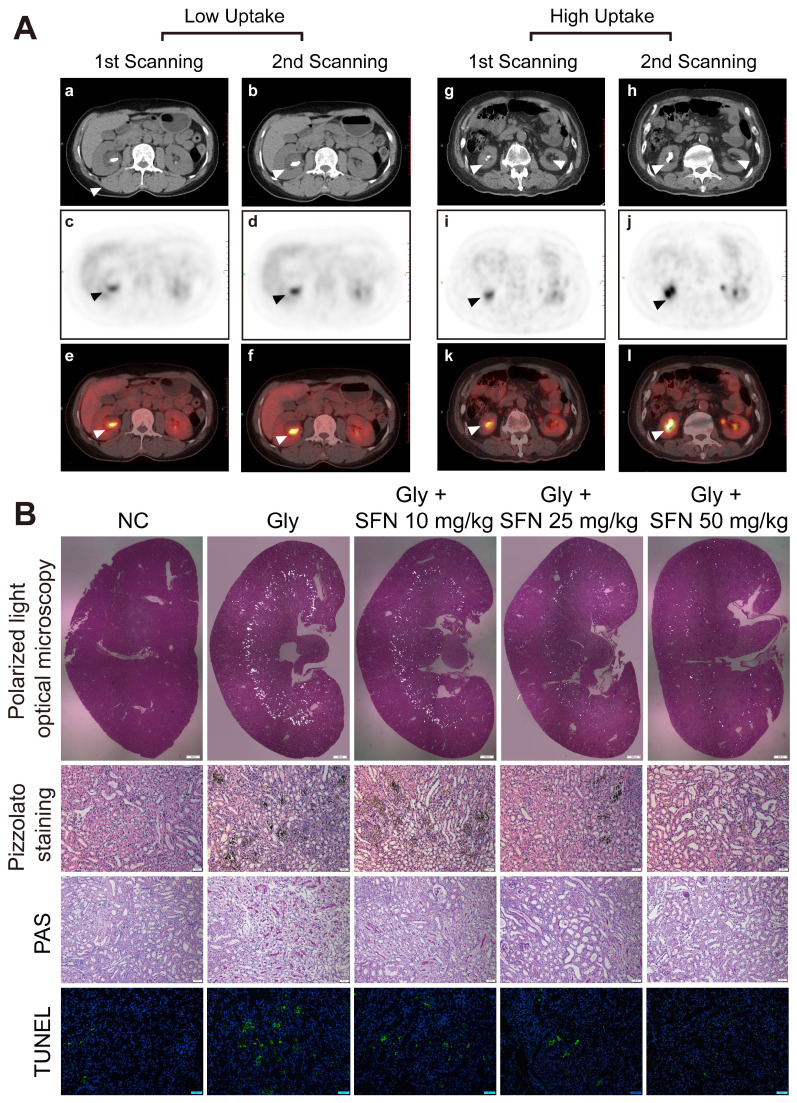
** Increasing inflammationin in rapidly progressive stone patients while decreasing kidney inflammatory injury found in SFN treated CaOx nephrocalcinosis mice. (A)** PET-CT images demonstrating kidney inflammatory responses. (a, b, g, h) axial-CT, (c, d, i, j) axial-PET, and (e, f, k, i) axial-fused PET-CT images showing increased ^18^F-FDG uptake within the kidneys of stone patients. The arrow indicates the focal accumulation of ^18^F-FDG as a ring around the kidney stone. **(B)** Renal CaOx-crystal deposition was detected by polarized-light optical microscopy of mice treated with increasing concentrations of SFN (20× magnification, scale bar: 500 µm). Pizzolato staining confirmed that CaOx crystal deposition mainly appeared in the corticomedullary junction area (200× magnification; scale bar: 20 µm). PAS staining detected renal tubular epithelial cell injury (200× magnification; scale bar: 20 µm). TUNEL staining revealed TECs death occurring in kidney tissues (200× magnification; scale bar: 50 µm). *P < 0.05; **P < 0.01, as determined by one-way ANOVA (B).

Kidney inflammatory injury and crystal deposition induced by CaOx are considered to be two pivotal steps in the development of kidney stone. Crystal nephropathy is associated with significant intrarenal inflammation that can cause acute kidney injury and enhance stone progression. We retrospectively analyzed 150 patients with lung cancer and who had been treated at Tongji Hospital, with 10 patients found to have kidney stones at initial PET-CT scanning. After 6 months, delayed imaging PET-CT analysis showed a significant increase in kidney stone size in patients with a higher ROI_max_ around the renal parenchyma. We also found that the inflammatory state in the kidney caused by stones appeared to affect the growth of the stones (Figure 1A, Table S2).

### SFN attenuates renal CaOx crystal deposition and CaOx nephrocalcinosis-induced kidney inflammatory injury* in vivo*

SFN is a promising drug for blocking inflammation. To investigate the protective effects of SFN on renal CaOx crystal-induced renal tubular epithelial cell injury and CaOx crystals deposition, mice were pre-treated with different doses of SFN (10 mg/kg, 25 mg/kg, or 50 mg/kg) for 3 days. Then, we established a Gly-induced kidney CaOx nephrocalcinosis mouse model with 75 mg/kg/day Gly intraperitoneal injection for 7 days (along with SFN treatment). Consistent with our clinical findings, polarized-light optical microscopy and Pizzolato staining revealed significantly decreased CaOx crystals deposition along with increased SFN concentration (Figure 1B, Figure S1A, B). Importantly, PAS and TUNEL staining showed that SFN attenuated CaOx nephrocalcinosis-induced inflammatory injury and death of TECs in a dose-dependent manner (Figures 1B, Figure S1C, D).

### Nrf2 significantly suppresses TLR4 and IRF1 levels in a mouse model of CaOx nephrocalcinosis

**Figure 2:**
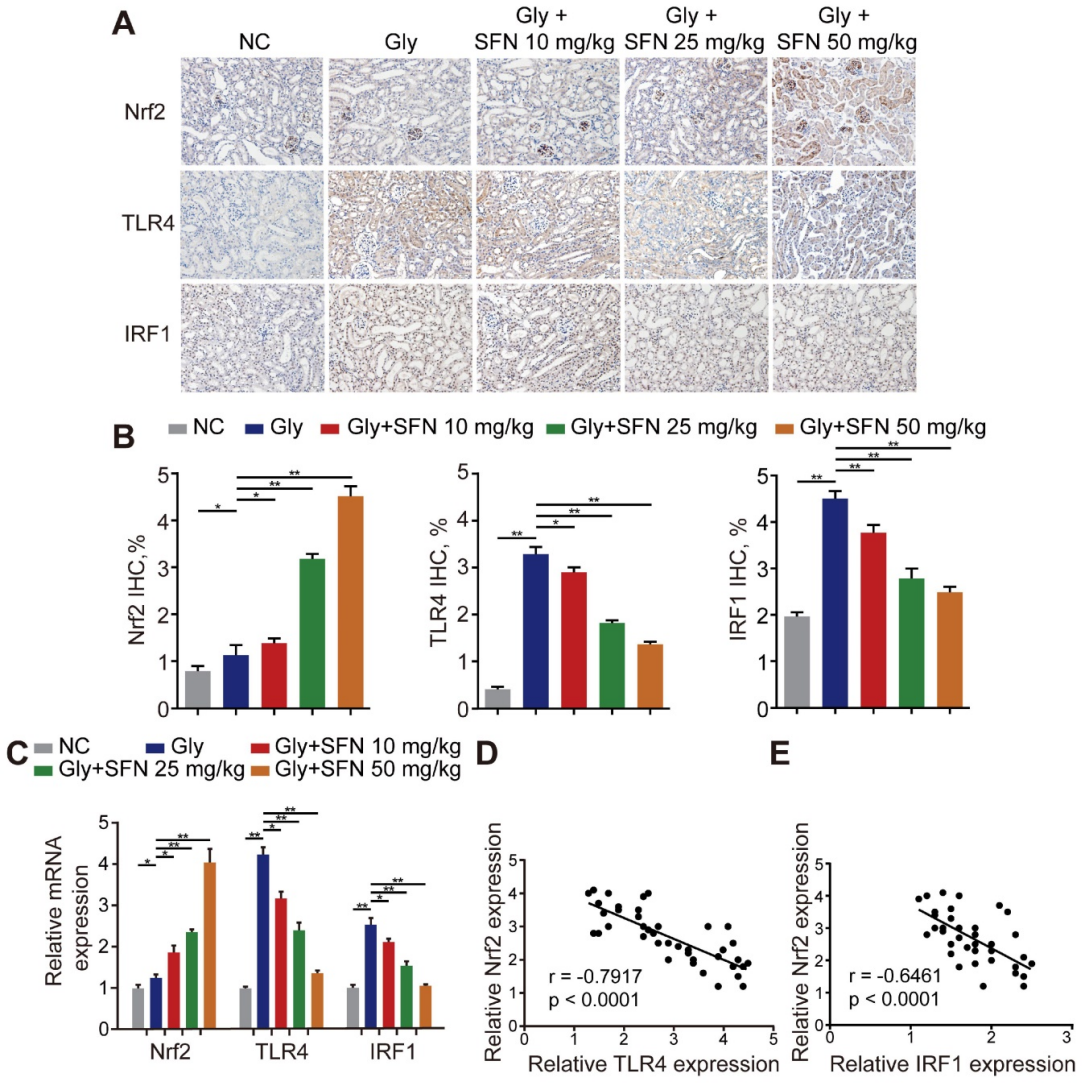
** Nrf2 significantly suppresses TLR4 and IRF1 levels in a mouse model of CaOx nephrocalcinosis. (A)** IHC staining of kidney Nrf2, TLR4, and IRF1 in CaOx nephrocalcinosis mice treated with SFN (400× magnification; scale bar: 40 µm). SFN treatment increases Nrf2 and inhibits the expression of TLR4 and IRF1 in CaOx nephrocalcinosis mouse tubular epithelial cells. **(B)** Quantification of IHC staining of Nrf2, TLR4, and IRF1 in SFN treated CaOx nephrocalcinosis mouse model. **(C)** qPCR detection of Nrf2, TLR4, and IRF1 expression in SFN-treated CaOx nephrocalcinosis mouse kidney samples and comparison with normal controls. **(D, E)** Pearson's correlation analysis of Nrf2 levels relative to TLR4 and IRF1. Data represent the mean ± standard error (SE) of three independent experiments. *P < 0.05; **P < 0.01, as determined by one-way ANOVA (B, C) or Pearson's correlation (D, E).

Because SFN is a pharmacological activator of Nrf2, we searched the Gene Expression Omnibus (GEO) database for gene-expression profiles and found one available GEO dataset (GSE71695) from a recent genome-wide, gene-expression profile analysis of BMDMs from Nrf2-WT and -knockout mice. RNA-seq analysis revealed that knocking out Nrf2 in BMDMs resulted in 301 upregulated genes and 680 downregulated genes (|Log_2_FC| ≥ 1; P < 0.05). The top 20 genes with the lowest P-values related to differential expression are shown in a volcano plot (Figure S2A, B). To further analyze the differentially expressed genes, Kyoto Encyclopedia of Genes and Genomes and Gene Ontology analyses were performed using Database for Annotation, Visualization and Integrated Discovery (david.ncifcrf.gov) and the keywords “inflammatory response,” “macrophage activation” and “toll-like receptor signaling”, for annotation clustering of pathway terms (Figure S2C, D). Based on our findings, we hypothesized that TLR4 and IRF1 might play crucial roles in inflammation-related diseases regulated by Nrf2. To test this hypothesis, we treated Gly-induced kidney CaOx nephrocalcinosis-model mice with increasing doses of SFN. We observed strong IHC staining of Nrf2 with decreased staining of TLR4 and IRF1 (two M1Mϕ-polarization regulators) in an SFN-dependent manner (Figure 2A, B). Additionally, we found that TLR4 and IRF1 expression clearly decreased, whereas Nrf2 increased in SFN-treated CaOx nephrocalcinosis mouse tubular epithelial cells (Figure 2C). We then examined their relationships by performing Pearson's correlation analysis, finding that Nrf2 level correlated negatively with TLR4 and IRF1 levels (Figure 2D, E). These data indicated that SFN treatment markedly decreased TLR4 and IRF1 levels in CaOx nephrocalcinosis-model mice and BMDMs, and that Nrf2 level negatively correlated with TLR4 and IRF1 levels.

### Nrf2 suppresses TLR4 and IRF1 levels and attenuates M1-macrophage polarization induced by supernatants from COM-stimulated TECs *in vitro*

**Figure 3:**
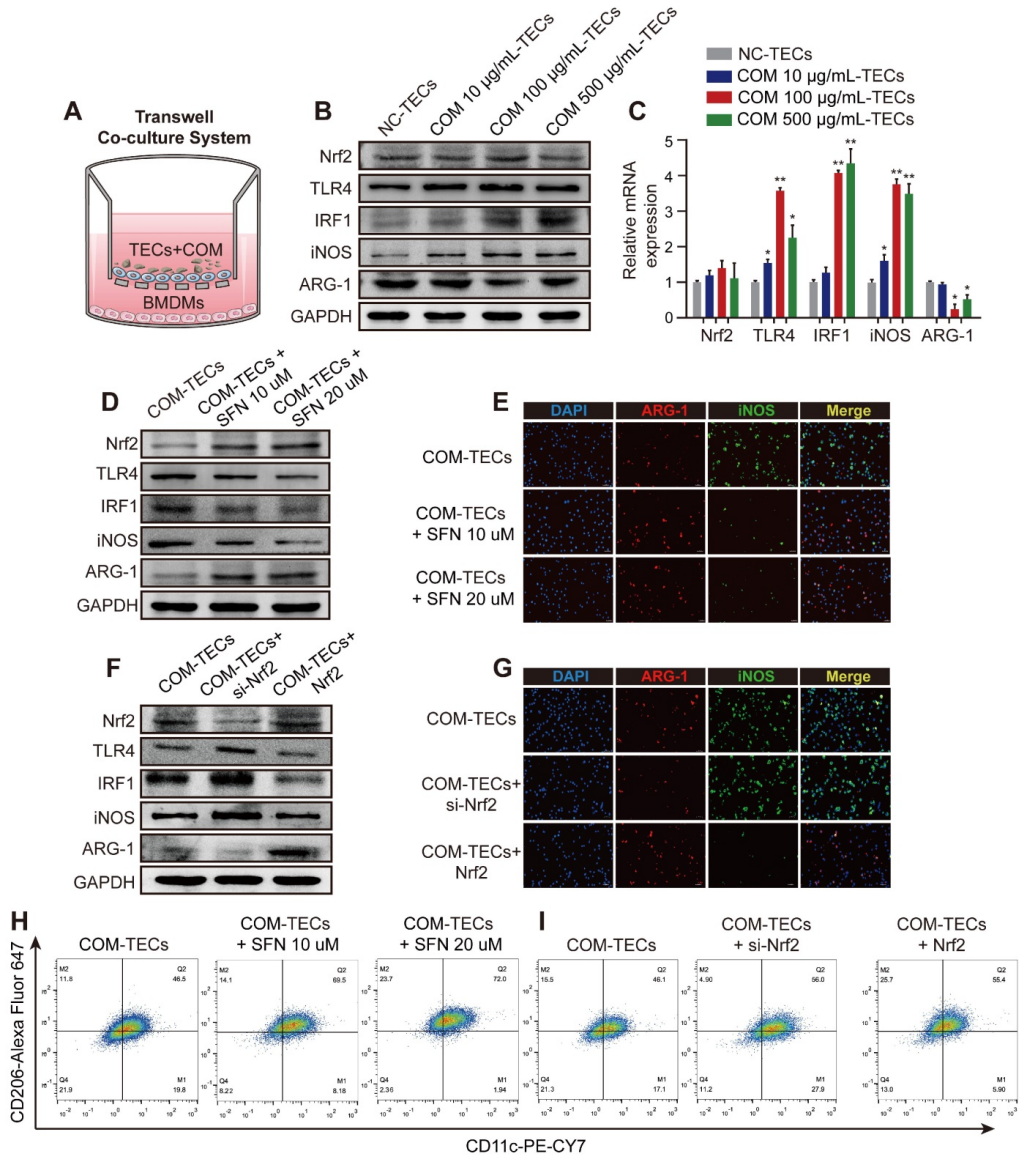
** Nrf2 suppresses TLR4 and IRF1 levels and promotes M2Mϕ polarization *in vitro*. (A)** BMDM and COM-TECs coculture schematic diagram. Western blot **(B)** and qPCR **(C)** analyses of Nrf2, TLR4, IRF1, iNOS, and ARG-1 levels in BMDMs co-cultured with increasing COM dose stimulated TECs. GAPDH was used as an internal control. **(D, F)** Western blot detection of Nrf2, TLR4, IRF1, iNOS, and ARG-1 levels following SFN treatment or Nrf2 upregulation/downregulation in BMDMs co-cultured with COM-stimulated TECs. GAPDH was used as an internal control. **(E, G)** The distribution of iNOS (green) and ARG-1 (red) in BMDMs according to immunofluorescence. **(H, I)** Flow cytometric analysis of the polarization state of BMDMs using anti-CD11c and anti-CD206 in F4/80^+^ and CD11b^+^ cells. The data are shown as the mean ± standard error (SE) of three independent experiments. *P < 0.05; **P < 0.01, as determined by Student's t test (C) or one-way ANOVA (E, G, H, I).

Exposure to supernatants from COM (dose-dependent) stimulated TECs activated the inflammatory response of BMDMs in the co-culture system (Figure 3A-C). After treated BMDMs with SFN (10 μM or 20 μM) in a co-culture system with COM-stimulated TECs. Figure 3D and Figure S3A show that SFN treatment activated Nrf2 expression, which then downregulated the expression of TLR4, IRF1, and iNOS (M1Mϕ-phenotype markers) and upregulated the expression of ARG-1 (an M2Mϕ-phenotype marker). Moreover, immunofluorescence staining using BMDMs revealed that SFN decreased iNOS and increased ARG-1 levels (Figure 3E, Figure S3C). Consistent with these results, flow cytometry analysis suggested that SFN promoted M2Mϕ polarization and suppressed M1Mϕ polarization (Figure 3H, Figure S3E). To verify the key role of SFN-activated Nrf2 in COM-TECs stimulated macrophage polarization, we transfected BMDMs with a Nrf2-overexpression vector and siNrf2. Consistent with previous results, Nrf2 and siNrf2 significantly decreased or promoted COM-TECs stimulated M1Mϕ polarization, respectively (Figures 3F, G, I, Figure S3B, D, F).

### Nrf2 inhibits TLR4 and IRF1 expression by directly controlling miR-93-5p

**Figure 4:**
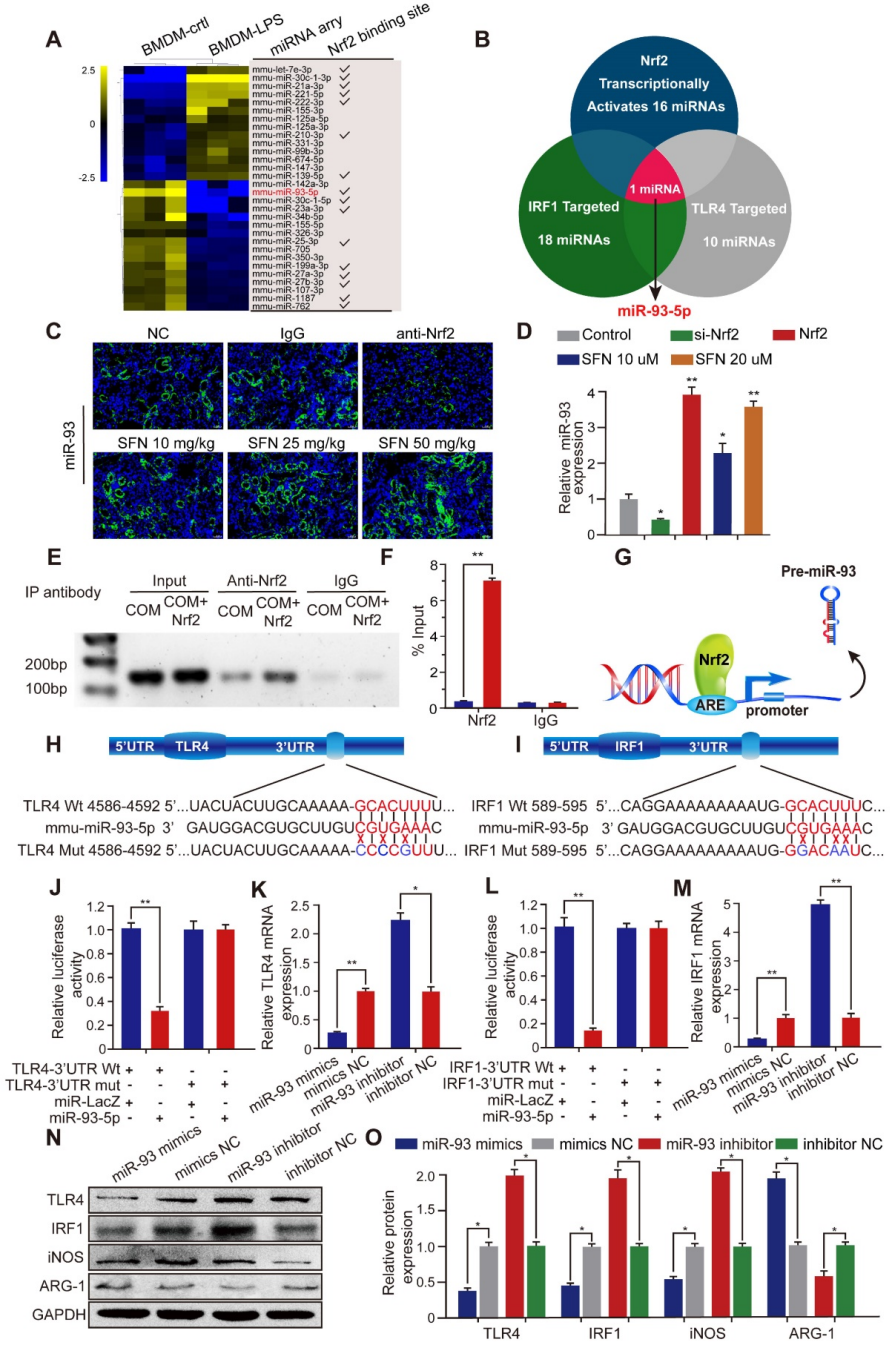
** Nrf2 inhibits TLR4 and IRF1 expression by directly binding to miR-93 promoter. (A)** miRNA-array heatmap revealed the top 30 LPS-regulated miRNAs in BMDMs. Additionally, miRNAs predicted to be transcriptionally activated by Nrf2 (based on the JASPRA database) are highlighted. **(B)** Venn analysis identified miRNAs that could target TLR4 and IRF1 and be transcriptionally activated by Nrf2. **(C)** Renal expression of mmu-miR-93-5p following treatment with an Nrf2-neutralizing antibody or SFN in mice with CaOx nephrocalcinosis was detected using FISH (200× magnification for all panels; scale bar: 20 µm). **(D)** qPCR analysis of the expression levels of mmu-miR-93-5p in BMDMs. U6 RNA was detected as an internal control. **(E, F)** ChIP assays and ChIP qPCR analysis of Nrf2 binding to the predicted miR-93 ARE in BMDMs treated with Nrf2. **(G)** A schematic model showing Nrf2 directly binds the promoter of miR-93 and activates its transcription. **(H, I)** Schematic representation of mutant and WT seed sequences of miR-93 targeting the 3' UTRs of TLR4 and IRF1. Luciferase reporters harboring putative target sites in WT and mutant 3' UTRs of TLR4 **(J)** or IRF1 **(L)** were co-transfected with miR-93 mimics (100 nM). TLR4 **(K)** and IRF1 **(M)** expression detected by qPCR in BMDMs transfected with miR-93 mimics or inhibitor. Western blot **(N)** and qPCR **(O)** detection of TLR4 and IRF1 levels, as well as the Mϕ-polarization markers iNOS and ARG-1 in BMDMs transfected with miR-93 mimics or inhibitor. GAPDH was used as an internal control. Data represent the mean ± standard error (SE) of three independent experiments. *P < 0.05; **P < 0.01, as determined by Student's t test (D, F) or one-way ANOVA (J-M, O).

Transcriptional activation miRNA is a major mechanism through which transcription factors exert their effects. To further investigate the mechanism by which Nrf2 regulates the progression of CaOx nephrocalcinosis, we searched the GEO datasets and found a lipopolysaccharide (LPS)-induced BMDM miRNA profile (GSE107095). Hierarchical clustering and heatmap analysis revealed the top 30 LPS-regulated miRNAs in BMDMs. Analysis using the JASPAR database (http://jaspar.genereg.net/) showed that 16 of these miRNAs were potentially transcriptionally activated by Nrf2 directly binding to their respective promoter (Figure 4A). Using the TargetScan, miRanda, miRWalk, and RNA22 databases, we identified 18 and 10 miRNAs predicted to target IRF1 and TLR4, respectively (Table S3). Venn analysis indicated that among the 16 miRNAs, only miR-93-5p can target TLR4 and IRF1 mRNA and be transcriptionally activated by Nrf2 (Figure 4B).

To determine whether SFN affects miR-93 expression, we determined miRNA-expression levels in mouse kidneys by FISH and in BMDMs by qPCR. The results demonstrated that SFN treatment induced miR-93 expression in a dose-dependent manner, whereas this was suppressed in mice treated with an Nrf2-neutralizing antibody and in BMDMs treated with siNrf2 (Figure 4C, D). To identify the mechanisms underlying the processes, we searched for putative antioxidant response elements (AREs) targeted by Nrf2 within a 2-kb region flanking the transcription start site of miR-93 (using the JASPRA database) and found an ARE consensus binding site (5′-TTCAATGAGTAGGCA-3′). We then performed a ChIP assay to test for binding between Nrf2 and the putative ARE, finding that Nrf2 binds to the ARE in the miR-93 promoter and induced its expression (Figure 4E, F). Figure 4G showed the direct binding to the miR-93 promoter by Nrf2.

Bioinformatics analysis showed that the 3′ UTR of both TLR4 and IRF1 contain conserved, putative miR-93-5p-targeting sites. We first constructed luciferase reporters in psiCHECK-2 plasmids containing either WT or mutated miR-93 target sequences in the 3′ UTRs of TLR4 or IRF1 (wt-TLR4, wt-IRF1 and mut-TLR4, mut-IRF1, respectively) (Figure 4H, I). Compared with the control group, the luciferase activities of the wt-TLR4 and wt-IRF1 vectors decreased significantly when co-transfected with miR-93 mimics, whereas no significant change was observed following co-transfection of the mut-TLR4 and mut-IRF1 vectors along with miR-93 mimics (Figure 4J, L). Consistently, qPCR and western blot revealed that transfecting miR-93 mimics or inhibitor decreased or increased TLR4 and IRF1 levels in BMDMs, respectively, both at the mRNA and protein levels (Figure 4K, 4M-4O).

### SFN regulates macrophage polarization and kidney inflammatory injury via the Nrf2-miR-93-TLR4/IRF1 pathway *in vitro*

**Figure 5:**
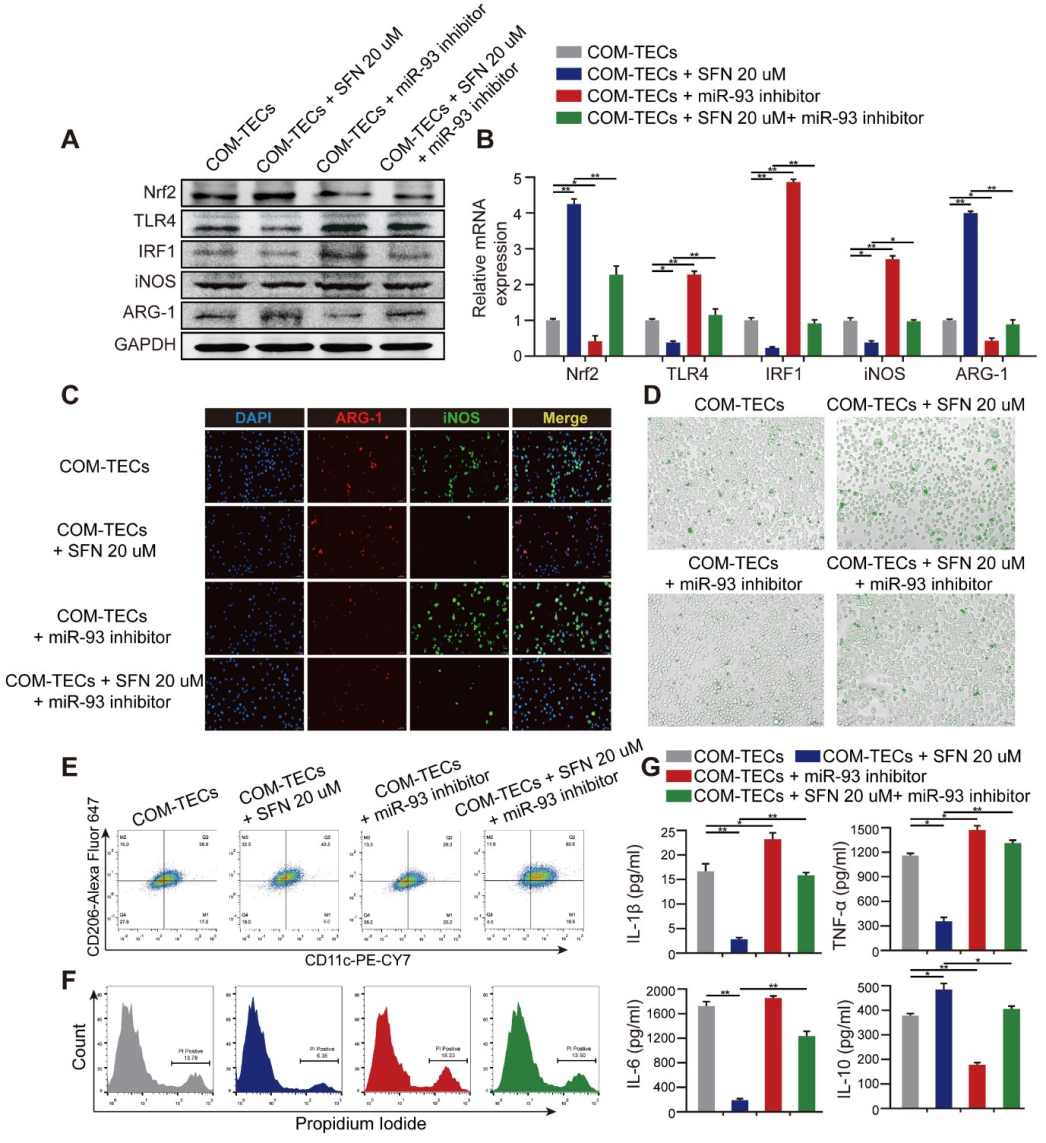
** SFN dependeds on the Nrf2-miR-93-TLR4/IRF1 axis to suppress TLR4 and IRF1 expression and promote M2Mϕ polarization *in vitro*.** Western blot **(A)** and qPCR **(B)** analyses of Nrf2, TLR4, IRF1, iNOS, and ARG-1 levels in BMDMs treated with SFN and/or miR-93 inhibitor. GAPDH was used as an internal control. **(C)** The distribution of iNOS (green) and ARG-1 (red) in BMDMs treated with SFN and/or miR-93 inhibitor according to immunofluorescence (200× magnification; scale bar: 20 µm). **(D)** Fluorescence microscopy analysis of BMDM phagocytic ability. COM crystals were labeled with an Alexa Fluor 488-conjugated IgG and directly cultured with treated BMDMs (200× magnification; scale bar: 20 µm). Flow cytometric analysis of BMDM polarization **(E)** and TECs necrosis **(F)** in co-cultured cells treated with SFN and/or miR-93 inhibitor. Data represent the mean ± standard error (SE) of three independent experiments. *P < 0.05; **P < 0.01, as determined by one-way ANOVA **(B-G).**

M2Mϕs are correlated with the resolution of inflammation and tissue regeneration. To determine whether SFN drives M2Mϕ polarization to suppress CaOx-induced TECs inflammation and eliminate crystal formation via the Nrf2-miR-93-TLR4/IRF1 axis *in vitro*, we treated BMDMs with miR-93 mimics and co-cultured them with COM-stimulated TECs. We found that expression of M1Mϕ markers (iNOS and CD11c) was significantly suppressed, whereas an M2Mϕ marker (ARG-1 and CD206) was augmented (Figure S4A-D). We then quantified the levels of proteins and mRNAs associated with the M1 or M2 phenotype. SFN treatment suppressed the M1 markers TLR4 and IRF1, which was prevented by treatment with the miR-93 inhibitor (Figure 5A, B). Interestingly, the miR-93 inhibitor suppressed expression of Nrf2. We hypothesize that there may be a positive feedback loop between Nrf2 and miR-93 which need further investigation. Consistently, immunofluorescence and flow cytometry analyses revealed that the miR-93 inhibitor reversed SFN-mediated promotion of M2Mϕ polarization (Figure 5C, E & Figure S5B, D). After treating BMDMs with or without SFN or miR-93 inhibitor treatment for 24 h in the COM-stimulated TECs co-culture system then directly culturing BMDMs with IgG Alexa Fluor 488-labeled COM for 5 h. Phagocytic ability of BMDMs was analyzed by using fluorescence microscopy (Figure S5A). As expected, BMDMs treated with SFN showed a higher rate of COM-crystal phagocytosis than the group treated with the miR-93 inhibitor (Figure 5D; Figure S5C).

The TECs-injury and -inflammatory profiles of Mϕ in response to SFN or miR-93 was further examined by flow cytometry and ELISA. We co-cultured BMDMs with COM-stimulated TECs, and following SFN or miR-93 treatment for 24 h, TECs necrosis was measured by flow cytometry. The results showed that SFN significantly reduced COM-induced TECs necrosis, whereas miR-93 inhibition reversed this effect (Figure 5F). Consequently, ELISAs to determine levels of proinflammatory and anti-inflammatory cytokines in co-culture media revealed suppressed levels of IL-1β, TNF-α, and IL-6, whereas IL-10 secretion increased in the SFN-treated group. Additionally, treating BMDMs with SFN and the miR-93 inhibitor showed that miR-93 inhibition partially reversed the anti-inflammatory effects of SFN (Figure 5G). These results suggested that SFN regulated Mϕ polarization, and attenuated CaOx induced kidney injury via the Nrf2-miR-93-TLR4/IRF1 pathway.

### SFN suppresses CaOx-crystal deposition and CaOx nephrocalcinosis-induced injury of renal tubular epithelial cells via the Nrf2-miR-93-TLR4/IRF1 axis *in vivo*

**Figure 6:**
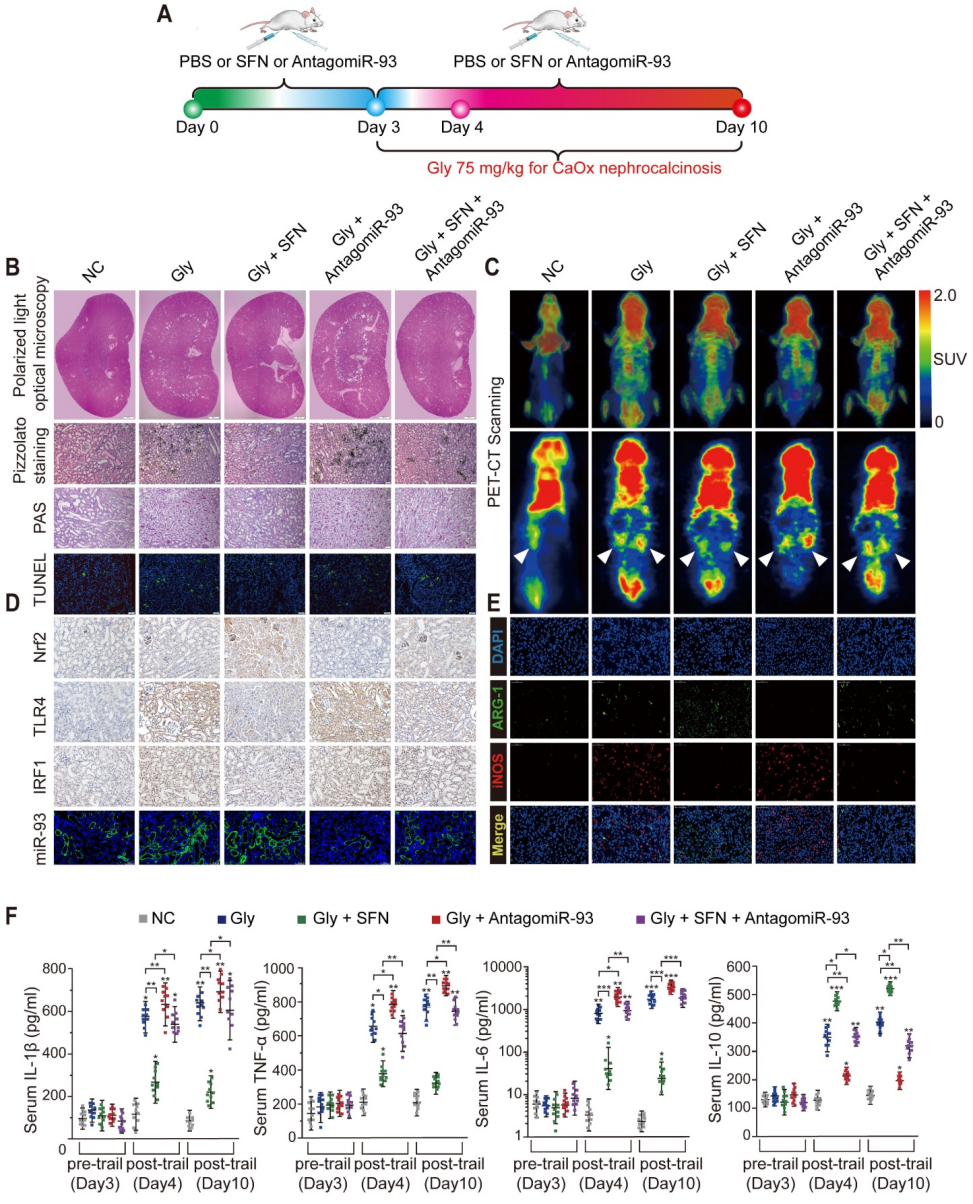
** SFN dependeds on the Nrf2-miR-93-TLR4/IRF1 axis to suppress CaOx crystal deposition and kidney injury *in vivo*. (A)** Diagram of the experimental design. **(B)** Polarized-light optical microscopy detection of renal CaOx crystal deposition in mice treated with SFN and/or antagomiR-93 (20× magnification; scale bar: 500 µm). Pizzolato staining to detect corticomedullary CaOx crystal deposition. PAS and TUNEL staining to detect renal tubular epithelial cell injury (200× magnification; scale bar: 20 µm). **(C)** ^18^F-FDG PET-CT scanning to detect kidney inflammation in CaOx nephrocalcinosis mice treated with SFN and/or antagomiR-93. **(D)** IHC detection of Nrf2, TLR4, and IRF1 levels (400× magnification; scale bar: 40 µm) and FISH detection of miR-93 expression in kidney tissue (200× magnification; scale bar: 20 µm). **(E)** The distributions of iNOS (red) and ARG-1 (green) in kidney tissues according to immunofluorescence. **(F)** Serum levels of the proinflammatory cytokines IL-1β, TNF-α, and IL-6 and the anti-inflammatory cytokine IL-10 according to ELISA on days 3, 4, and 10. *P < 0.05; **P < 0.01, as determined by one-way ANOVA (B-F).

Renal CaOx-crystal deposition was associated with diffuse Mϕ infiltration and tubular necrosis. To further explore the mechanism by which SFN suppresses CaOx-crystal deposition and CaOx-induced kidney injury *in vivo*, we pretreated mice with SFN and/or antagomiR-93 for 3 days and used Gly to establish a CaOx nephrocalcinosis mouse model for further treatment (Figure 6A). Polarized-light optical microphotography and Pizzolato staining revealed that SFN significantly decreased CaOx crystal deposition in mouse kidneys, but that antagomiR-93 had the opposite effect. PAS staining and TUNEL assays confirmed that antagomiR-93 decreased the effect of SFN in protecting against renal tubular epithelial cell injury (Figures 6B, Figure S6B-E). To assess local inflammatory responses in the kidneys, we determined ^18^F-FDG uptake to assess the extent of inflammation. Micro PET-CT scanning showed that SFN notably alleviated ^18^F-FDG uptake and the SUV_max_ of kidneys, whereas antagomiR-93 increased these parameters in CaOx nephrocalcinosis mice (Figure 6C). Accordingly, strong IHC staining for Nrf2 and miR-93 (FISH) and weak IHC staining for intestinal TLR4 and IRF1 were found in mice treated with SFN (Figure 6D, Figure S6F-I). Consistent with IHC data, qPCR showed low TLR4, IRF1 and iNOS when treated with SFN, whereas AntagomiR-93 reversed this effect (Figure S6A).

SFN treatment robustly suppressed Gly-induced kidney inflammation and necrosis, possibly due to its effects on Mϕ polarization. As expected, immunofluorescence and IHC assays revealed a worsened kidney interstitial inflammatory response with increased infiltration of M1Mϕ (iNOS) in the nephrocalcinosis mouse model after intraperitoneal administration of antagomiR-93 as compared with the results of SFN treatment (Figure 6E, Figure S6J, Figure S7). Furthermore, we analyzed levels of proinflammatory cytokines (IL-1β, IL-6, and TNF-α), anti-inflammatory cytokine (IL-10), and serum creatinine and BUN levels in order to determine acute inflammatory responses and kidney functions after different treatments in our mouse model. After 3 days of SFN or antagomiR-93 treatment, we observed elevated levels of inflammatory cytokines and decreased renal function on day 1 of Gly injection. However, SFN significantly protected renal function from Gly and promoted IL-10 secretion, whereas antagomir-93 had the opposite effect. On the final observation day, we observed slight improvements in renal function in the SFN-treated group, whereas the antagomir-93 group showed continual deterioration of renal function and release of a large number of proinflammatory cytokines (Figure 6F, Table S4). These results suggested that SFN might promote M2Mϕ phagocytic capacity to clan up CaOx crystal, and suppress CaOx nephrocalcinosis-induced injury to renal tubular epithelial cells depended on the Nrf2-miR-93-TLR4/IRF1 pathway.

## Discussion

Urinary stone formation is a complex process involving multiple factors. The mechanisms of stone formation involve CaOx crystallization, crystal growth, aggregation and adhesion to renal tubular epithelial cells have been extensively studied during the last few decades 5. Previously studies reveled that supersaturation of calcium, oxalate ions and acidic urine pH will promote crystallization and determine types of calcium oxalate crystals 1, 7, 44, 46. Meanwhile, CaOx crystallization, growth, and cell adhesion may be regulated by the action of modifiers and inhibitors, which range from small ions and molecules and proteins like citrate, Tamm-Horsfall glycoprotein, nephrocalcin and aspartic acid-rich protein superfamily 47-50. Anders HJ et.al also reported that TNFR signaling is of great important for CaOx crystal adhesion while proteins such as immunglobulin G and pentraxin 3 are the endogenous inhibitor of calcium oxalate crystal growth 4, 51, 52. Adherent crystals will ultimately form crystal plugs obstructing tubules followed by interstitial inflammation and renal injury 7, 53. The precise molecular mechanisms underlying crystal-related kidney injury and inflammation in the progression of kidney stone remain obscure.

Clinical data and our previous experimental findings show that exposure of kidney tubular epithelial cells to CaOx crystals can generate excess reactive oxygen species, thereby causing inflammation and inducing renal injury 34, 54, 55. Recent studies show that Mϕs participate in the progression of kidney interstitial-crystal deposition, and that proinflammatory M1Mϕ accelerates kidney injury and renal crystal development, whereas anti-inflammatory M2Mϕ suppresses renal crystal formation and deposition by phagocytosing CaOx crystals and inhibiting oxidation-induced kidney injury 23, 56-58. In the present study, we discovered that SFN activated Nrf2 to attenuate kidney inflammatory injury and suppress CaOx crystal deposition along with increasing M2Mϕ infiltration in mouse kidneys. It is now widely recognized that SFN exerts its cytoprotective, and anti-inflammatory functions via Nrf2 activation 59. Yan et al. reported that Nrf2 played a protective role against lung injury induced by intestinal ischemia-reperfusion (I/R) by modulating TLR4 and Akt signaling 60. Additionally, Xi et al. demonstrated that sirtuin-3 reduced the adhesion of CaOx crystals in TECs by activating the Nrf2/heme oxygenase-1-signaling pathway 61. Although data from several studies have revealed that Nrf2 plays a pivotal role in inflammation-related tissue injury and regulating Mϕ polarization 27, 28, 62, the precise mechanisms by which Nrf2 suppresses CaOx nephrocalcinosis by regulating Mϕ polarization remains unidentified. Here, we found that Nrf2 played a key role in Mϕ polarization regulation, which decreased renal inflammatory injury and crystal deposition in the pathogenesis of CaOx-induced nephrocalcinosis.

TLR4 and IRF1 have been identified as two powerful regulators of Mϕ proliferation, differentiation, and polarization. A pathogenic role for these two proteins has been demonstrated in many diseases, including renal injury. Poluzzi et al. showed that interfering with the interaction between biglycan and TLR4 co-receptors increased M2Mϕ levels and represented a promising therapeutic intervention to curtail renal I/R injury 63. Lv et al. demonstrated that inhibiting TLR4 or NF-κB suppressed LPS-induced Mincle expression on macrophages, as well as IL-1β, TNF-α, and IL-6 production, suggesting a novel therapy for acute kidney injury associated with M1Mϕ 64. NF-κB also binds to the IRF1 promoter, and Eckhardt et al. reported that concomitant knockdown of IRF1 and NF-κB p65 can inhibit cell apoptosis and proinflammatory cytokine production 65. Consistent with these findings, we showed that Nrf2 downregulated TLR and IRF1 levels via miR-93 to promote a phenotypic switch from M1Mϕ to M2Mϕ and reduce proinflammatory cytokine levels, which subsequently protected against CaOx-induced nephrocalcinosis injury.

MiRNAs are molecular switches that play pivotal roles in regulating Mϕ macrophage polarization and kidney injury 66, 67. Li et al. reported that miR-146a promoted M2Mϕ polarization and diminished M1Mϕ polarization in systemic juvenile idiopathic arthritis by targeting inhibin subunit beta A 68. Pan and colleagues reported that miR-21 conferred anti-inflammatory effects against sepsis-induced acute kidney injury by inhibiting the PDCD4/NF-κB and PTEN signaling pathways in the kidney 69. Additionally, Qi et al. provided evidence indicating that miRNA let-7i adjusted LPS-induced renal inflammatory injury caused by the TLR4/MyD88 signaling pathway 70. Moreover, miR-93 is reportedly a crucial regulator of inflammation. Ma et al. revealed that miR-93 negatively regulates NF-κB signaling by binding to secreted phosphoprotein 1, which reduced the injury and inflammatory responses of mouse cardiac microvascular endothelial cells 71. Furthermore, upregulation of miR-93 reduced inflammatory cytokine expression and alleviated neuropathic pain development by targeting signal transducer and activator of transcription 3 in rats with injury owing to chronic constriction of the sciatic nerve 72. In the present study, miR-93 demonstrated a dual effect of promoting M2Mϕ polarization and inhibiting TECs inflammatiory injury caused by CaOx crystals.

In summary, we described the protective role of SFN in CaOx nephrocalcinosis-induced injury of renal tubular epithelial cells and Gly-induced kidney CaOx-crystal deposition. Additionally, we revealed the mechanism by which Nrf2-miR-93 interaction regulates the TLR4/IRF1 pathway. Our study provides new mechanistic insights into Mϕ polarization regulation in CaOx nephrocalcinosis disease and might aid the development of novel therapeutic strategies targeting renal CaOx crystal deposition and reducing crystal-induced renal inflammatory injury.

## Supplementary Material

Supplementary figures and tables.

## Abbreviations

CaOx: calcium oxalate
Mϕ: macrophage
SFN: sulforaphane
Nrf2: nuclear factor erythroid 2-related factor 2
TLR4: Toll-like receptor 4
NF-κB: nuclear factor kappaB
iNOS: inducible nitric oxide synthase
IL: interleukin
IRF1: interferon regulatory factor 1
TNF: tumor necrosis factor
Gly: glyoxylate acid
PBS: phosphate-buffered saline
BMDMs: bone marrow-derived macrophages
TECs: renal tubular epithelial cells
COM: calcium oxalate monohydrate
miRNA: microRNA
siRNA: small-interfering RNA
HE: hematoxylin-eosin
PAS: periodic acid-Schiff
TUNEL: terminal deoxynucleotidyl transferase dUTP nick-end labeling
IHC: immunohistochemistry
BUN: blood urea nitrogen
FISH: fluorescent in situ hybridization
PET-CT: positron emission tomography computed tomography
SUV: standardized uptake value
ROI: region of interest
ELISA: enzyme-linked immunosorbent assay
qPCR: quantitative polymerase chain reaction
GAPDH: glyceraldehyde 3-phosphate dehydrogenase
ARG-1: arginase 1
DAPI: 4,6-diamidino-2-phenylindole
FITC: fluorescein isothiocyanate
ChIP: chromatin Immunoprecipitation
UTR: untranslated region
WT: wild type
ANOVA: analysis of variance
GEO: Gene Expression Omnibus
LPS: lipopolysaccharide
AREs: antioxidant response elements
