# Sulforaphane elicts dual therapeutic effects on Renal Inflammatory Injury and crystal deposition in Calcium Oxalate Nephrocalcinosis: Erratum

**DOI:** 10.7150/thno.68830

**Published:** 2022-01-01

**Authors:** Haoran Liu, Xiaoqi Yang, Kun Tang, Tao Ye, Chen Duan, Peng Lv, Libin Yan, Xiaoliang Wu, Zhiqiang Chen, Jianhe Liu, Yaoliang Deng, Guohua Zeng, Jinchun Xing, Zhangqun Ye, Hua Xu

**Affiliations:** 1 Department of Urology, Tongji Hospital, Tongji Medical College, Huazhong University of Science and Technology, Wuhan, China.; 2 Department of Urology, The Second Affiliated Hospital of Kunming Medical University, Kunming, China.; 3 Department of Urology, The First Affiliated Hospital of Guangxi Medical University, Nanning, China.; 4 Department of Urology, The First Affiliated Hospital of Guangzhou Medical University, Guangzhou, China.; 5 Department of Urology, The First Affiliated Hospital of Xiamen University, Xiamen, China.

The authors apologize for the original version of our paper unfortunately contained an incorrect representative image of immunohistochemical (IHC) staining for IRF1. Noting that 200x IHC images in the first submitted version were correct. For presenting high resolution images according to one of the reviewer's suggestion, we assembled 400x IHC images in our revised manuscript. IHC staining for IRF1 in the group of Gly + SFN 25 mg/kg was misused as Gly + SFN 50 mg/kg. We apologize that at the time of figure assembly, we choose representative images by mistake. We apologize for our careless at the time of figure assembly. We confirm that it would not affect any results and conclusions of the paper. The correct representative images for Figure 2A are shown below. The authors apologize for any inconvenience that this error may have caused.

## Figures and Tables

**Figure 1:**
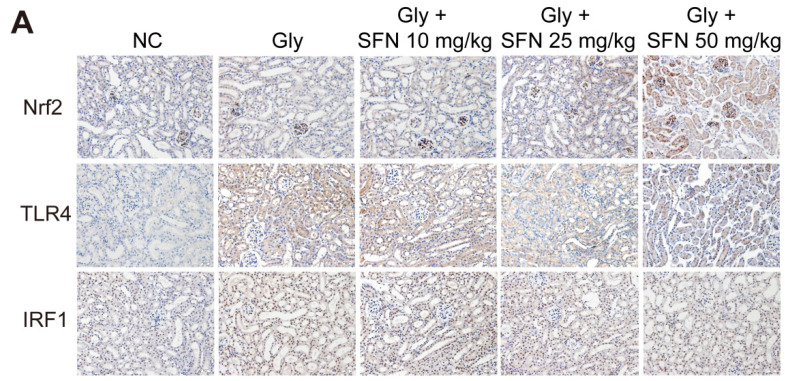
Corrected image for original Figure 2A.

