# Supplementary material for: Sulforaphane elicts dual therapeutic effects on Renal Inflammatory Injury and crystal deposition in Calcium Oxalate Nephrocalcinosis
Source: Theranostics. 2020 Jun 5;10(16):7319–34. doi: 10.7150/thno.44054 (PMC7330860; doi:10.7150/thno.44054)
Supplement: Supplementary file 1 — Supplementary figures and tables. [file thnov10p7319s1.pdf]

Supplemental Materials

Supplementary Table 1. List of primer sequences used for the real-time qPCR analysis.

Supplementary Table 2. PET-CT examination reveals relationship between intrarenal inflammation and kideny stone size.

Supplementary Table3. Selected miRNAs targeted TLR4 or IRF1 positively predicted by four databases.

Supplementary Table 4. Serum BUN and Creatinine levels in mouse model.

Supplementary Figure 1. Quantification of CaOx crystal deposition, PAS staining, TUNEL staining in SFN treated CaOx nephrocalcinosis mice.

Supplementary Figure 2. RNA-Seq heatmap, Volcano plots, Kyoto Encyclopedia of Genes and Genomes enrichment and Gene ontology analyses in Nrf2 knockout BMDM.

Supplementary Figure 3. Quantification of real-time PCR (qPCR), immunofluorescence and flow cytometric analysis in BMDMs.

Supplementary Figure 4. Immunofluorescence and flow cytometry analysis the polarization of BMDMs transfected with miR-93 mimics or inhibitor.

Supplementary Figure 5. Quantification of immunofluorescence, phagocytosis rate and flow cytometric analysis in BMDMs.

Supplementary Figure 6. qPCR analyses of Nrf2, TLR4, IRF1, iNOS, ARG-1 levels in mouse kidney and quantification of CaOx crystal deposition, PAS staining, TUNEL staining, Nrf2, TLR4, IRF1 IHC staining, miR-93 FISH and immunofluorescence in mice kidney samples.

Supplementary Figure 7. IHC detection and quantification of F4/80 and iNOS levels in kidney samples.

Supplementary Table 1. List of primer sequences used for the real-time qPCR analysis

**Name**	**(5'-3')**	**SEQUENCE (5'-3')**
Nrf2	Forward	TTTTCCATTCCCGAATTACAGT
	Reverse	AGGAGATCGATGAGTAAAAATGGT
TLR4	Forward	TCTGGGGAGGCACATCTTCT
	Reverse	AGGTCCAAGTTGCCGTTTCT
IRF1	Forward	TTGGCATCATGGTGGCTGT
	Reverse	AAGGAGGATGGTCCCCTGTTT
iNOS	Forward	CACCTTGGAGTTCACCCAGT
	Reverse	ACCACTCGTACTTGGGATGC
ARG1	Forward	TGGCTTGCGAGACGTAGAC
	Reverse	GCTCAGGTGAATCGGCCTTTT
GAPDH	Forward	TGCTGAGTATGTCGTGGAGTCTA
	Reverse	AGTGGGAGTTGCTGTTGAAGTCG

Supplementary Table 2. PET-CT examination reveals relationship between intrarenal inflammation and kideny stone size.

Patients	ROImax (stone kidney)		Stone Volume (mm^3)	
	1st Scanning	2nd Scanning	1st Scanning	2nd Scanning
Patient NO.1	4.69	5.22	683.4	997.2
Patient NO.2	5.13	4.93	536.7	689.1
Patient NO.3	2.85	3.13	508.3	529.3
Patient NO.4	5.73	5.00	683.8	723.1
Patient NO.5	5.24	4.30	896.3	913.6
Patient NO.6	2.85	3.79	489.7	763.1
Patient NO.7	3.30	3.22	672.9	697.3
Patient NO.8	4.02	4.59	537.1	721.6
Patient NO.9	4.46	3.95	581.2	603.8
Patient NO.10	3.49	4.07	483.6	662.8

Abbreviations: ROI, region of interest.

Supplementary Table 3. Selected miRNAs targeted TLR4 or IRF1 positively predicted by four databases.

Gene	RefseqID	miRNA	MIMATid	miRWalk	miRanda	RNA22	Targetscan	SUM
TLR4	NC_000070.6	mmu-miR-182-5p	MIMAT0000211	1	1	1	1	4
TLR4	NC_000070.6	mmu-miR-216a-5p	MIMAT0000662	1	1	1	1	4
TLR4	NC_000070.6	mmu-miR-17-5p	MIMAT0000649	1	1	1	1	4
TLR4	NC_000070.6	mmu-miR-20a-5p	MIMAT0000529	1	1	1	1	4
TLR4	NC_000070.6	mmu-miR-20b-5p	MIMAT0003187	1	1	1	1	4
TLR4	NC_000070.6	mmu-miR-93-5p	MIMAT0000540	1	1	1	1	4
TLR4	NC_000070.6	mmu-miR-106a-5p	MIMAT0000385	1	1	1	1	4
TLR4	NC_000070.6	mmu-miR-106b-5p	MIMAT0000386	1	1	1	1	4
TLR4	NC_000070.6	mmu-miR-27a-3p	MIMAT0004633	1	1	1	1	4
TLR4	NC_000070.6	mmu-miR-27b-3p	MIMAT0004522	1	1	1	1	4
IRF1	NC_000077.6	mmu-miR-140-3p.1	MIMAT0000152	1	1	1	1	4
IRF1	NC_000077.6	mmu-miR-130a-3p	MIMAT0016983	1	1	1	1	4
IRF1	NC_000077.6	mmu-miR-130b-3p	MIMAT0004583	1	1	1	1	4
IRF1	NC_000077.6	mmu-miR-301a-3p	MIMAT0017008	1	1	1	1	4
IRF1	NC_000077.6	mmu-miR-301b-3p	MIMAT0017232	1	1	1	1	4
IRF1	NC_000077.6	mmu-miR-23a-3p	MIMAT0017019	1	1	1	1	4
IRF1	NC_000077.6	mmu-miR-23b-3p	MIMAT0016980	1	1	1	1	4
IRF1	NC_000077.6	mmu-miR-9-5p	MIMAT0000142	1	1	1	1	4
IRF1	NC_000077.6	mmu-miR-383-5p	MIMAT0000748	1	1	1	1	4
IRF1	NC_000077.6	mmu-miR-17-5p	MIMAT0000649	1	1	1	1	4
IRF1	NC_000077.6	mmu-miR-20a-5p	MIMAT0000529	1	1	1	1	4
IRF1	NC_000077.6	mmu-miR-20b-5p	MIMAT0003187	1	1	1	1	4
IRF1	NC_000077.6	mmu-miR-93-5p	MIMAT0000540	1	1	1	1	4
IRF1	NC_000077.6	mmu-miR-106a-5p	MIMAT0000385	1	1	1	1	4
IRF1	NC_000077.6	mmu-miR-106b-5p	MIMAT0000386	1	1	1	1	4
IRF1	NC_000077.6	mmu-miR-203-3p	MIMAT0000236	1	1	1	1	4
IRF1	NC_000077.6	mmu-miR-124-3p	MIMAT0000134	1	1	1	1	4
IRF1	NC_000077.6	mmu-miR-142a-3p	MIMAT0000155	1	1	1	1	4

miRWalk: http://zmf.umm.uni-heidelberg.de/apps/zmf/mirwalk2/generetsys-self.html

miRanda: http://www.microrna.org/microrna/getGeneForm.do

RNA22: https://cm.jefferson.edu/rna22/Interactive/

Targetscan: http://www.targetscan.org/vert_71/

Supplementary Table 4. Serum BUN and Creatinine levels in mouse model.

Parameter	Time	NC	Gly	Gly + SFN	Gly + AntagomiR-93	Gly + SFN + AntagomiR-93
Serum creatinine (mg/dl)	3rd day	0.156 ± 0.021	0.162 ± 0.018	0.161 ± 0.023	0.148 ± 0.016	0.152 ± 0.020
	4th day	0.157 ± 0.018	0.329 ± 0.028 [a]	0.241 ± 0.022 [b]	0.362 ± 0.030 [b]	0.362 ± 0.031 [c]
	10th day	0.159 ± 0.026	0.539 ± 0.043 [a]	0.228 ± 0.034 [b]	0.598 ± 0.03 [b]	0.326 ± 0.023 [c]
Serum BUN (mg/dl)	3rd day	23.36 ± 1.02	24.08 ± 1.13	23.65 ± 1.28	24.16 ± 1.02	22.96 ± 1.36
	4th day	23.85 ± 1.29	53.26 ± 3.39 [a]	42.68 ± 2.79 [b]	60.08 ± 3.49 [b]	49.36 ± 1.99 [c]
	10th day	22.26 ± 1.21	68.73 ± 4.89 [a]	35.99 ± 2.08 [b]	73.26 ± 4.04 [b]	43.76 ± 2.83 [c]

Abbreviations: BUN, blood urea nitrogen; NC, normal control; Gly, glyoxylate.

[a] p <0.05 compare with the NC group

[b] p <0.05 compare with the Gly group

[c] p <0.05 compare with the Gly + SFN group

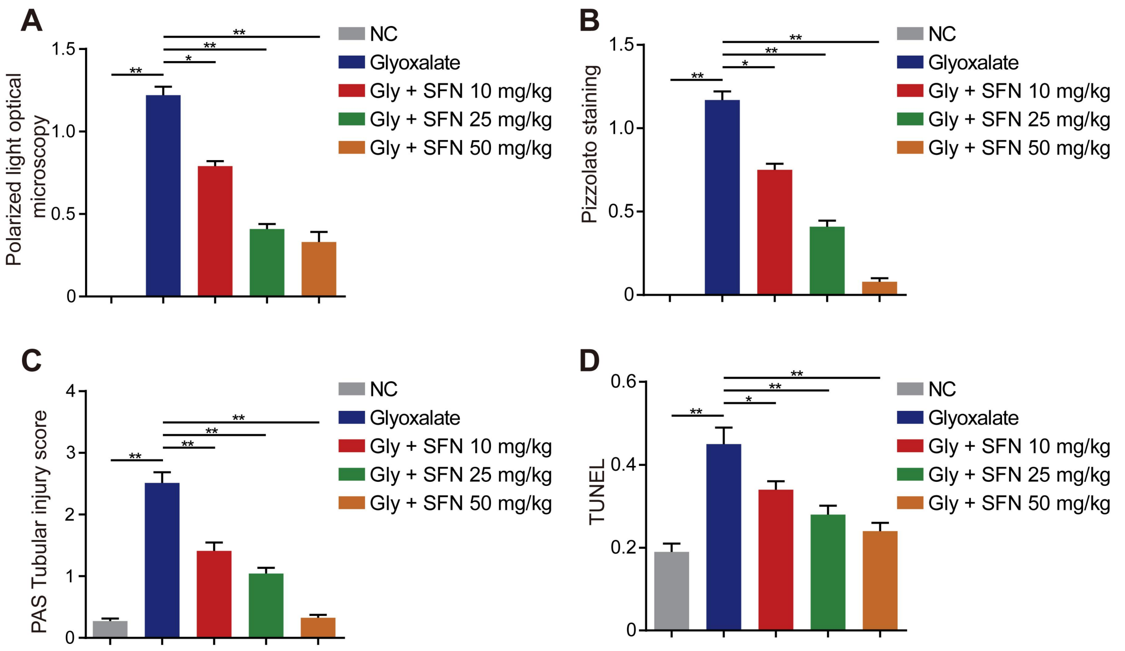

Supplementary Figure 1. Quantification of CaOx crystal deposition, PAS staining, TUNEL staining in SFN treated CaOx nephrocalcinosis mice. (A) The ratio of areas with renal crystal deposition detected by polarized light optical microscopy. (B) The ratio of areas with kidney corticomedullary junction area crystal deposition. (C) The percentage of injured renal tubules displayed in PAS staining. (D) The average number of TUNEL-positive cells per high power field (200×; n=10 fields per section). The data were showed as mean±SD of three independent experiments. *P < 0.05; **P < 0.01, by one-way ANOVA (A-D).

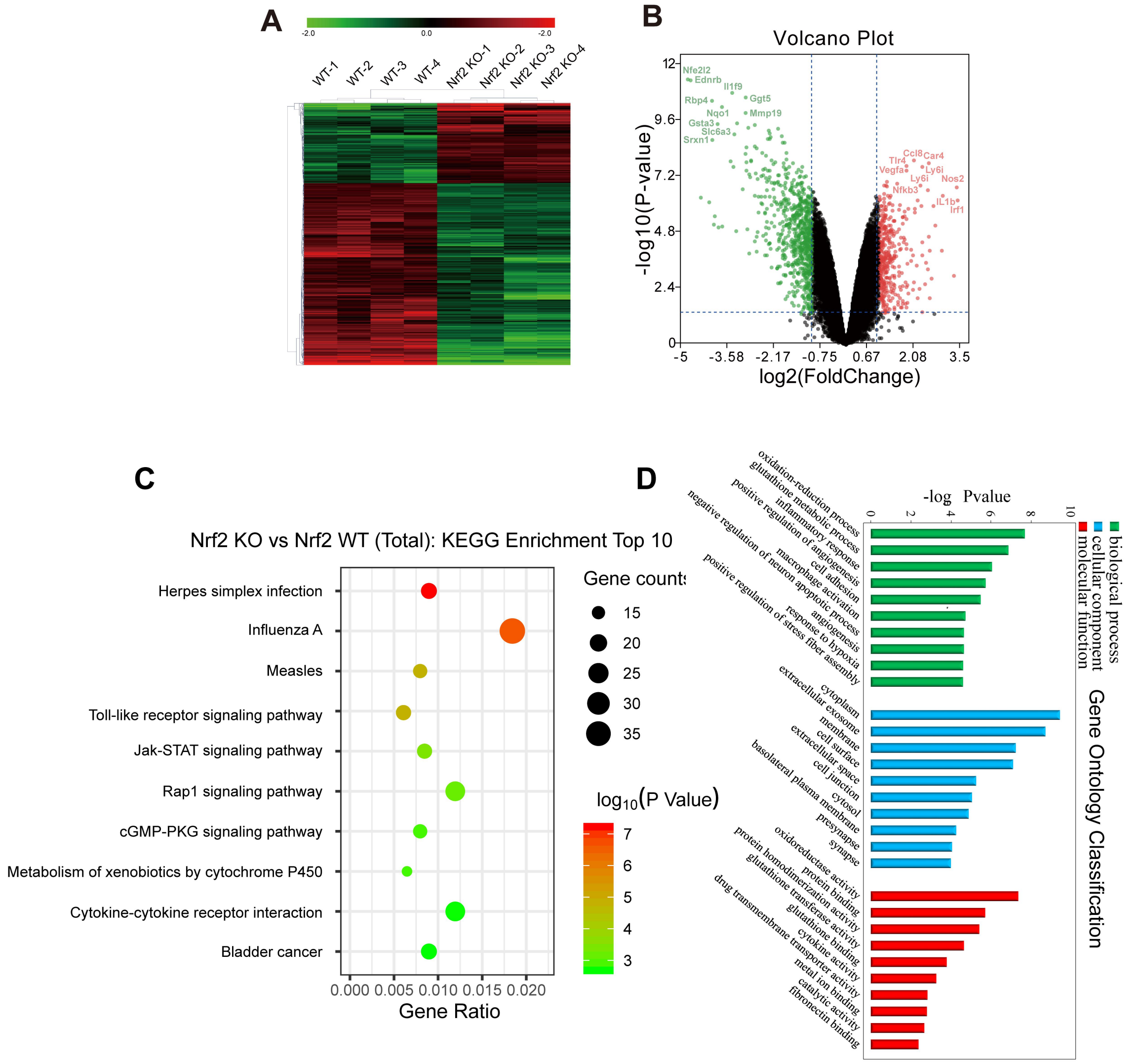

Supplementary Figure 2. RNA-Seq heatmap, Volcano plots, Kyoto Encyclopedia of Genes and Genomes enrichment and Gene ontology analyses in Nrf2 knockout BMDM.

(A) RNA-Seq heatmap showing significantly altered mRNAs in the BMDMs of Nrf2-knockout mice. (B) Volcano plots showing differentially expressed mRNA transcripts between WT and Nrf2-knockout BMDMs. (C) Kyoto Encyclopedia of Genes and Genomes enrichment analyse showed the top 10 significant pathway in Nrf2 knockout BMDM, in which Toll-like receptor signaling pathway containing TLR4 and Jak-STAT signaling pathway containing IRF1. (D) Gene ontology analyse showed that inflammatory response and macrophage activation play a vital role in Nrf2 knockout BMDM.

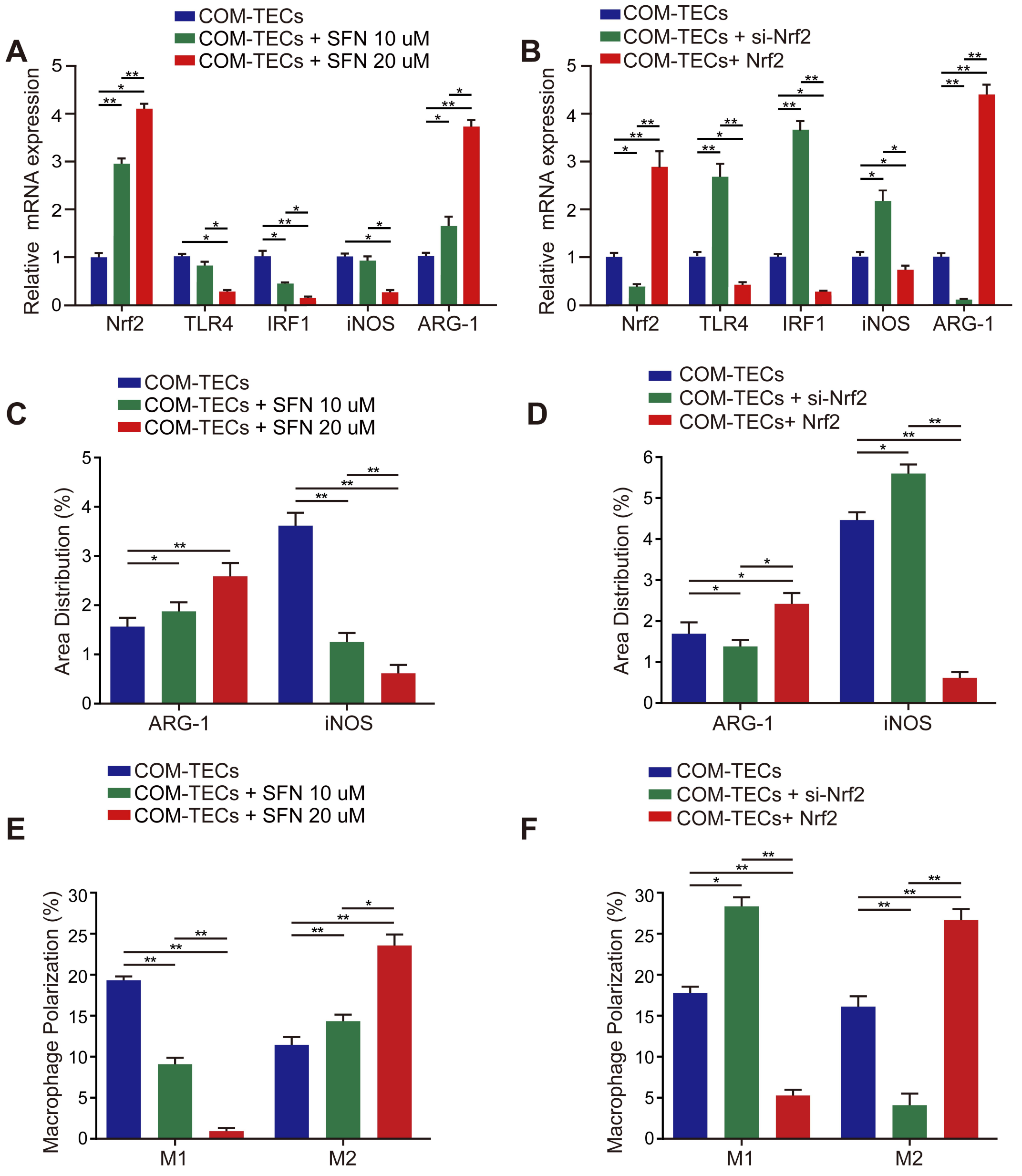

Supplementary Figure 3. Quantification of real-time PCR (qPCR), immunofluorescence and flow cytometric analysis in BMDMs. (A, B) Real-time quantitative PCR (qPCR) analysis of Nrf2, TLR4, IRF1, iNOS, and Arg1 expression in BMDMs. GAPDH was used as an internal control. (C, D) Quantification of ARG-1 and iNOS fluorescent of Figure 3E, G. (E,F) Quantification of macrophage polarization of Figure 3H, I.The data were showed as mean±SD of three independent experiments. *P < 0.05; **P < 0.01, by one-way ANOVA (A-F).

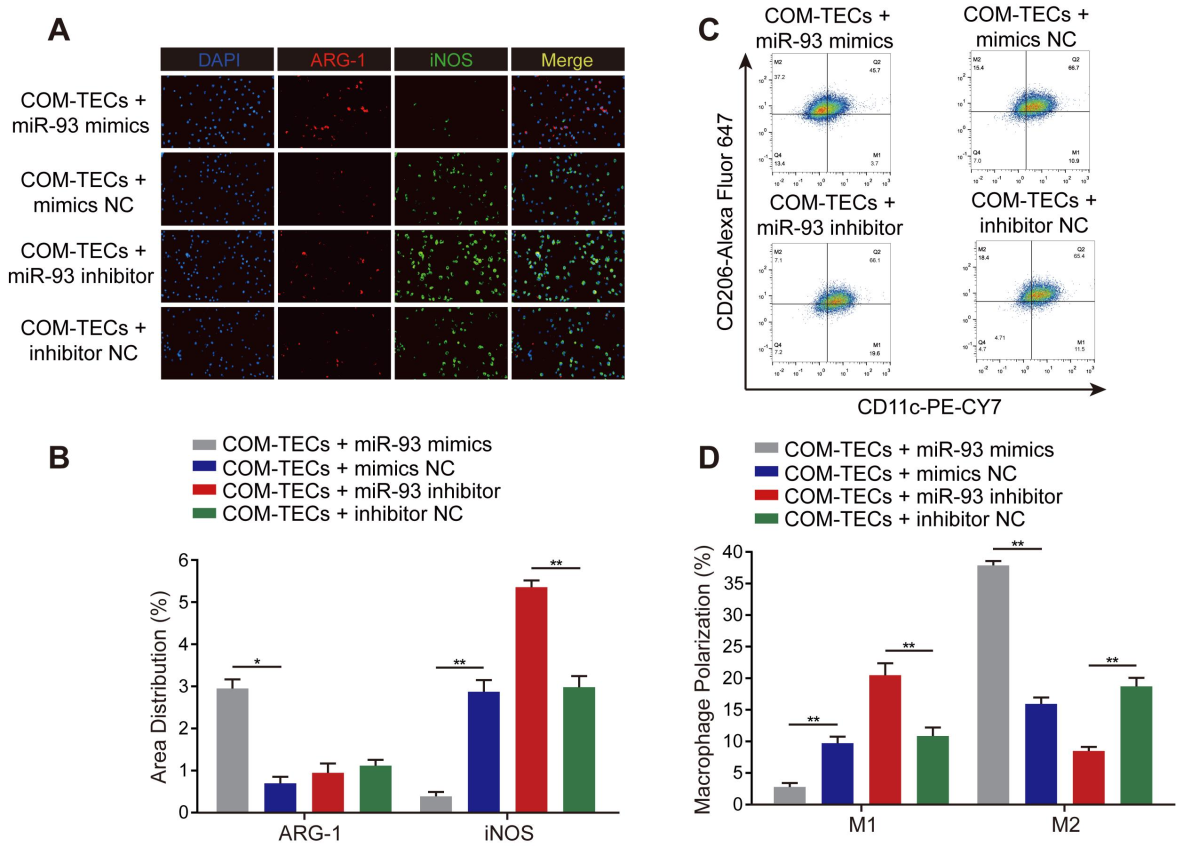

Supplementary Figure 4. Immunofluorescence and flow cytometry analysis the polarization of BMDMs transfected with miR-93 mimics or inhibitor. (A,B) The distribution and quantification of iNOS (green) and Arg1 (red) in BMDMs was detected by Immunofluorescence assay (C, D) Flow cytometry analyzed and quantification of BMDMs polarization state with anti-CD11c and CD206 in stored F4/80$^+$ and CD11b$^+$ cells. The data were showed as mean±SD of three independent experiments. *P < 0.05; **P < 0.01, by one-way ANOVA (A-D).

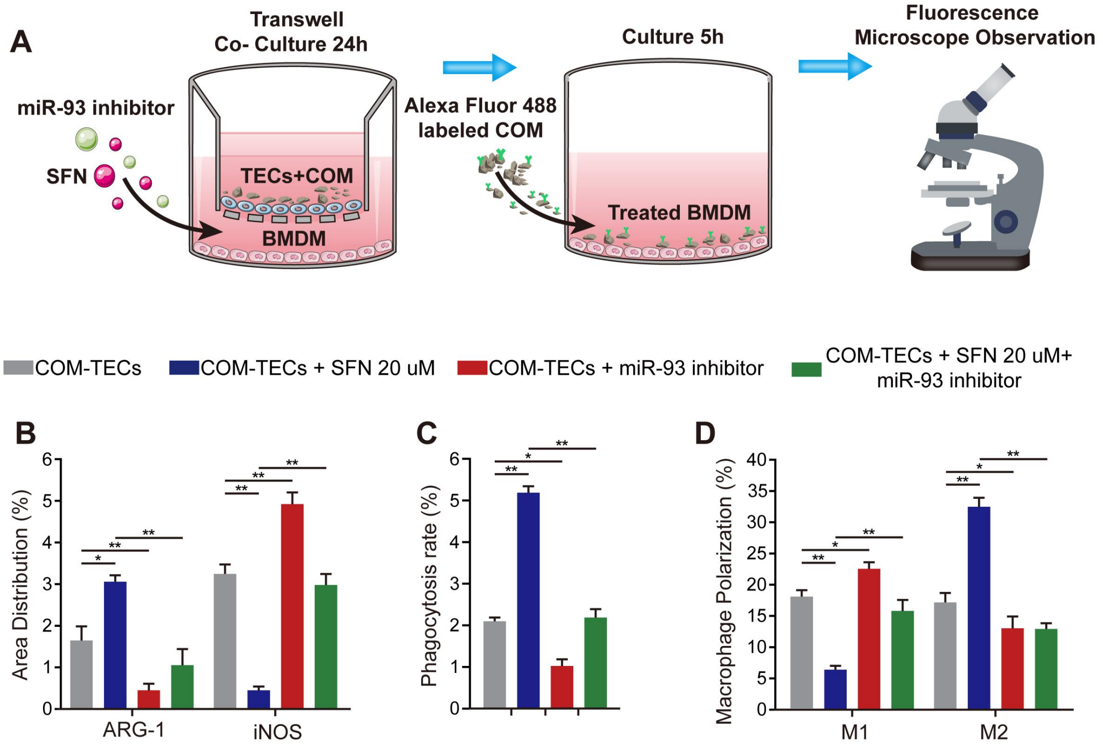

Supplementary Figure 5. Quantification of immunofluorescence, phagocytosis rate and flow cytometric analysis in BMDMs. (A) Schematic diagram of BMDMs phagocytic capacity testing. (B) Quantification of ARG-1 and iNOS fluorescent of Figure 5C. (C) Quantification of macrophage phagocytosis rate of Figure 5D. Quantification of macrophage polarization of Figure 5E. The data were showed as mean±SD of three independent experiments. *P < 0.05; **P < 0.01, by one-way ANOVA (B-D).

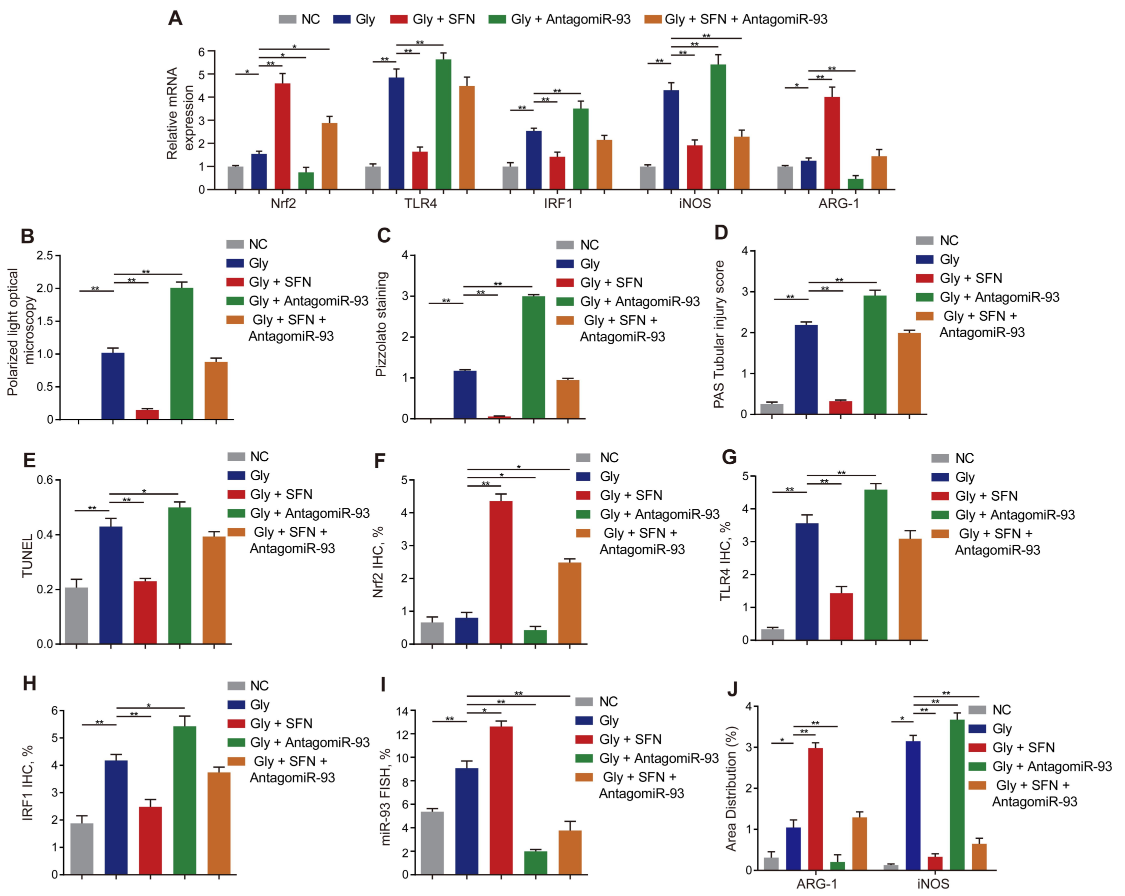

Supplementary Figure 6. qPCR analyses of Nrf2, TLR4, IRF1, iNOS, ARG-1 levels in mouse kidney and quantification of CaOx crystal deposition, PAS staining, TUNEL staining, Nrf2, TLR4, IRF1 IHC staining, miR-93 FISH and immunofluorescence in mice kidney samples. (A) qPCR detection of Nrf2, TLR4, IRF1, iNOS and ARG-1 expression in SFN-treated CaOx nephrocalcinosis mouse kidney samples and comparison with normal controls. (B) The ratio of areas with renal crystal deposition detected by polarized light optical microscopy. (C) The ratio of areas with kidney corticomedullary junction area crystal deposition. (D) The percentage of injured renal tubules displayed in PAS staining. (E) The average number of TUNEL-positive cells per high power field (200×; n=10 fields per section). (G, H, I) The ratio of areas with positive expression of Nrf2, TLR4, and IRF1 in IHC. (H) The ratio of areas with positive expression of miR-93 in FISH. (J) Quantification of ARG-1 and iNOS fluorescent. The data were showed as mean±SD of three independent experiments.

*P < 0.05; **P < 0.01, by one-way ANOVA (A-I).

Supplementary Figure 7. IHC detection and quantification of F4/80 and iNOS levels in kidney

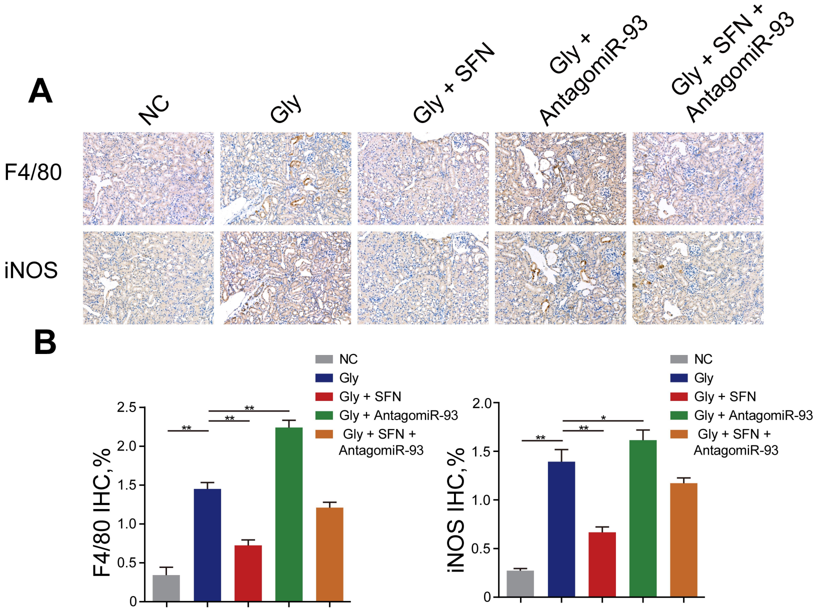

samples. (A) IHC staining of F4/80 and iNOS expression in kidney tissue (200× magnification; scale bar: 20 μm). (B) The ratio of areas with positive expression of F4/80 and iNOS expression in kidney tissue. The data were showed as mean±SD of three independent experiments. *P < 0.05; **P < 0.01, by one-way ANOVA (A, B).
